# Magneto‐X Effects in Magnetic Soft Materials and Their Applications

**DOI:** 10.1002/advs.202523234

**Published:** 2026-02-21

**Authors:** Ziyin Xiang, Xiangling Xia, Benjamin Ducharne, Yujie Ye, Jie Shang, Run‐Wei Li

**Affiliations:** ^1^ YuYao Innovation Institute Zhejiang Wanli University Ningbo China; ^2^ Ningbo Institute of Materials Technology and Engineering Chinese Academy of Sciences Ningbo China; ^3^ College of Materials Science and Opto‐Electronic Technology University of Chinese Academy of Sciences Beijing China; ^4^ LyTMaX IRL3757 CNRS Univ Lyon INSA Lyon Centrale Lyon Université Claude Bernard Lyon 1 Tohoku University Sendai Japan; ^5^ LGEF INSA‐Lyon University Lyon Villeurbanne France; ^6^ Ningbo Institute of Digital Twin Ningbo China; ^7^ Eastern Institute of Technology Ningbo China

**Keywords:** flexible electronics, magnetic soft materials, magneto‐responsive effects, soft robotics

## Abstract

Magnetic soft materials (MSMs) represents an emerging class of composite materials that integrate magnetic responsiveness with the mechanical compliance of soft polymers, gels, and fluids. This review systematically summarizes the fundamental magneto‐responsive effects—including magnetorheological, magnetoelastic, magnetothermal, magneto‐driven deformation, magnetoresistive, and magnetoelectric effects—and classifies MSMs by matrix and filler type. It highlights recent multidisciplinary advancements in soft robotics, biomedical engineering, and flexible electronics, demonstrating their capabilities in untethered actuation, targeted therapy, and self‐powered sensing. Finally, the review addresses persistent challenges such as multi‐physics modeling and scalable fabrication, while outlining a future roadmap toward intelligent, integrated systems. This work provides a comprehensive reference for advancing the science and application of MSMs across multiple fields.

## Graphical abstract

This review systematically explores magnetic soft materials (MSMs), a novel class of composites that transform under magnetic fields. It catalogs fundamental “Magneto‐X” effects, classifies materials by their matrix and magnetic fillers, and highlights transformative applications in soft robotics, biomedical devices, flexible electronics, etc. Finally, it addresses key challenges in modeling and manufacturing, outlining a roadmap towards intelligent, integrated systems.

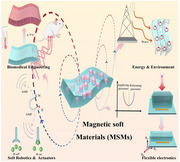

## 1 Introduction

Magnetic soft materials (MSMs) is an emerging class of composite material systems that combine the responsive properties of magnetic materials with the mechanical compliance of soft substances [1, 2, 3]. It typically consists of magnetic fillers (such as magnetic particles) dispersed within a soft polymer matrix (such as liquids, gels, or elastomers) [4, 5, 6]. “Magnetic soft materials” is fundamentally distinct from “soft magnetic materials.” The term “soft” in “soft magnetic materials” refers to their magnetic properties, namely high magnetic permeability and low coercivity, which allow them to be easily magnetized and demagnetized [7, 8]. For example, iron, nickel, cobalt, and their alloys—such as Permalloy (Fe‐Ni) and Permendur (Iron‐Cobalt‐Vanadium)—all belong to soft magnetic materials. Under the influence of a magnetic field, the magnetic moment orientation of soft magnetic materials can change rapidly and easily, characterized by a narrow hysteresis loop and low coercivity [9]. In this paper, “Magnetic soft materials” specifically refers to composite materials composed of a soft matrix, reflecting their nature as engineered material systems. This term is used interchangeably in the broader literature with the physics‐oriented term ‘magnetic soft matter,’ which emphasizes their compliance and response. The term “soft” here emphasizes the mechanical properties of the material, specifically its overall flexibility and deformability. The matrix of most MSMs—such as magnetic elastomers and hydrogels—exhibits a Young modulus typically ranging from 10 kPa to 10 MPa, which is orders of magnitude lower than that of conventional functional materials (often > GPa) [10, 11]. These soft matrices can sustain large reversible deformations, commonly on the scale of hundreds of micrometers to several millimeters, corresponding to strains generally exceeding 10% under practical loading conditions [11, 12]. As a composite material, the fillers in MSMs can be either hard magnetic materials or soft magnetic materials [13, 14], This provides the field with significant flexibility in material design [15].

Compared to traditional rigid magnetic materials, one of the significant advantages of magnetically controlled shape memory alloys is their ability to achieve much larger deformations, while retaining their responsiveness, programmability, and biocompatibility. These characteristics collectively form the foundation for its applications across multiple cutting‐edge fields [16]. In terms of responsiveness, traditional magnetic materials typically generate fixed magnetic moments or achieve macroscopic movement through magnetic field induction, but their deformation capabilities are limited. In contrast, MSMs can achieve complex and dynamic shape changes, such as bending, twisting, stretching, and folding. These deformation modes can be controlled remotely and contact‐free by adjusting the strength, direction, or frequency of the external magnetic field [17, 18, 19]. In terms of programmability, MSMs surpasse the simple attraction or repulsion behaviors of traditional magnets, enabling complex and programmable shape changes [2, 20, 21, 22]. By designing the type, concentration, and spatial distribution of magnetic fillers, combined with advanced manufacturing techniques such as 3D printing and magnetic field‐induced alignment, complex and time‐dependent deformations of MSMs can be achieved [2, 23, 24, 25]. The high coercivity of hard magnetic fillers allows the material to retain pre‐programmed magnetic polarity, thereby enabling nonlinear and high‐degree‐of‐freedom deformation control [26, 27]. In terms of biocompatibility, MSMs is fabricated using soft polymers such as hydrogels [28], silicone rubber, and polydimethylsiloxane (PDMS) [29]. These matrix materials possess excellent biocompatibility, and their mechanical properties are similar to those of biological tissues, significantly enhancing the safety of magnetic materials in biomedical applications. This makes them highly promising for use in the biomedical field, for instance, as drug delivery carriers, biosensors, or soft surgical instruments [19, 30].

The properties and applications of MSMs are primarily dictated by two interdependent design elements: the soft matrix and the embedded magnetic fillers. Their combination can be systematically understood through a dual‐level classification. First, The matrix material determines the material's foundational mechanical behavior and deformation scale. Based on the matrix material, they can be categorized into magnetic fluids, magnetic hydrogels and magnetic elastomers. Magnetic Fluids encompass magnetorheological (MR) fluids and ferrofluids, where magnetic particles are suspended in a Newtonian or non‐Newtonian liquid carrier (e.g., oil, water). The fluid matrix enables drastic, rapid, and reversible changes in rheological properties (like viscosity and yield stress) under a magnetic field but does not support sustained elastic deformation [31]. Magnetic Hydrogels comprising a cross‐linked hydrophilic polymer network (e.g., alginate, polyacrylamide) swollen with water and magnetic fillers, these materials combine magnetic responsiveness with high biocompatibility and tunable swelling/deswelling behavior. They are particularly promising for biomedical applications such as drug‐eluting scaffolds or soft actuators in physiological environments [32]. As for magnetic Elastomers, the magnetic particles are embedded within a cross‐linked rubbery polymer (e.g., silicone rubber, polyurethane). The elastic matrix allows for large, recoverable shape changes and provides a stable platform for programming complex magnetization patterns, making them the workhorse material for magnetic soft robots and reconfigurable structures [33]. Then, the type of filler dictates how the material interacts with and retains magnetic fields, which is directly linked to the achievable “Magneto‐X” effects. MSMs can be classified based on the type of magnetic fillers into soft magnetic fillers, hard magnetic fillers, and functional nanoscale fillers. For soft magnetic fillers, materials with low coercivity and high permeability (e.g., carbonyl iron powder, Mn‐Zn ferrites) are easily magnetized by and align with an external field. They are predominantly used in MR fluids and elastomers to achieve effects like the magnetorheological and giant magnetoelastic effects, where rapid, reversible microstructural rearrangement under a field leads to macroscopic property changes [34, 35, 36, 37, 38, 39]. For hard magnetic fillers: Permanent magnets with high coercivity and remanence (e.g., NdFeB, Sr‐ferrite microparticles) retain a programmed magnetization direction. They are essential for embedding complex magnetization patterns into elastomers and some gels, serving as the cornerstone for achieving programmable, magnetically driven deformation without a need for a continuous external field [1, 2, 22, 40, 41]. For functional nanoscale fillers: Biocompatible iron oxide nanoparticles (e.g., Fe_3_O_4_, γ‐Fe_2_O_3_) are often incorporated into hydrogel matrices for biomedical applications, leveraging their magnetothermal effect for hyperthermia therapy or imaging [42, 43]. Core‐shell structures (e.g., Fe@SiO_2_) are designed to enhance chemical stability and prevent adverse filler‐matrix interactions [5, 44]. This matrix‐filler interplay provides a vast design space. For instance, an innovative material like Liquid Metal‐based Magnetic Fluids emerges by selecting a liquid metal (e.g., EGaIn) as the fluid matrix, combining the MR effect with high electrical conductivity [45, 46, 47]. Ultimately, the strategic combination of a specific matrix with a tailored filler type enables the precise manifestation of the desired “Magneto‐X” effects discussed in the following sections.

**FIGURE 1:**
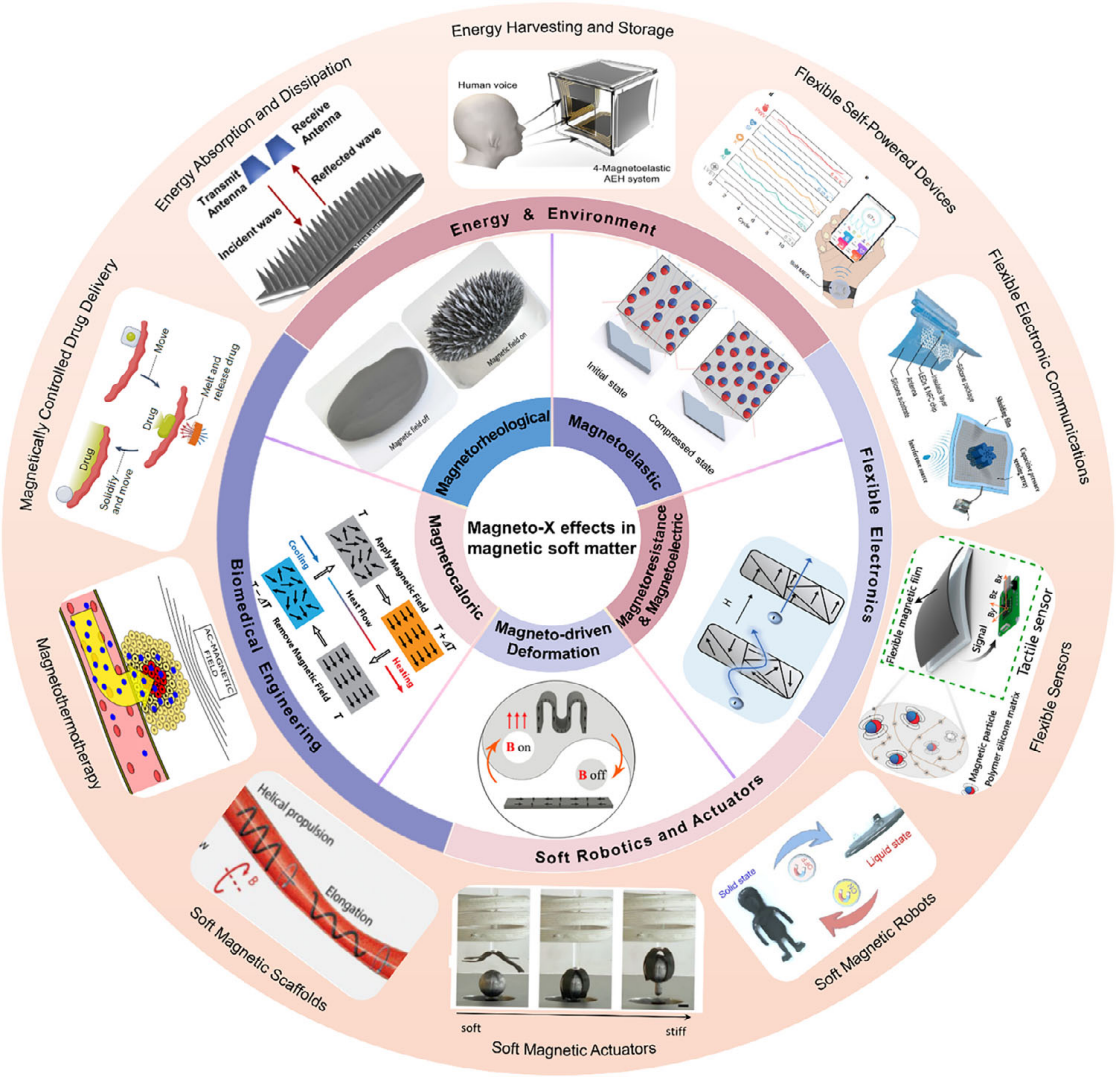
Magnetic soft materials, leveraging various magnetic response effects such as magnetorheological Adapted with permission [48]. Copyright 2018, Science Advances, magnetoelastic Adapted with permission [49]. Copyright 2021, Nature Materials, magnetoresistance & magnetoelectric Adapted with permission [50]. Copyright 2023, Advanced Physics Research, magneto‐driven deformation Adapted with permission [51]. Copyright 2021, ACS Applied Materials & Interfaces, and magnetocaloric effects, represent an emerging class of intelligent materials. Owing to their unique magnetic responsiveness, exceptional flexibility, and programmability, they demonstrate transformative application potential across fields including soft robotics and actuators, flexible electronics, energy and environment, and biomedical engineering. Specific implementations encompass magnetic soft actuators Adapted with permission [15]. Copyright 2019, Advanced Materials, magnetic soft robots Adapted with permission [52]. Copyright 2022, ACS Applied Materials & Interfaces, flexible sensors Adapted with permission [53]. Copyright 2022, ACS Nano, flexible electronic communications Adapted with permission [54, 55]. Copyright 2023, Nano Engergy, Copyright 2025, Advanced Science, flexible self‐powered devices Adapted with permission [49]. Copyright 2021, Nature Materials, energy harvesting and storage Adapted with permission [56]. Copyright 2025, Nature Materials, energy absorption and dissipation Adapted with permission [57]. Copyright 2023, Journal of Alloys and Compounds, magnetically controlled drug delivery Adapted with permission [45]. Copyright 2024, Nature Reviews Materials, magnetothermotherapy Adapted with permission [58]. Copyright 2022, Nano Materials and MSMs‐based magnetic scaffolds Adapted with permission [59]. Copyright 2024, Science Robotics.

MSMs, as an emerging class of intelligent materials, demonstrate transformative application potential in fields such as soft robotics, biomedicine, flexible electronics, and energy and environmental technologies, owing to their unique magnetic responsiveness and exceptional flexibility and programmability, the overall structure of this paper is shown in Figure 1. The field is currently undergoing a profound transition from fundamental scientific discoveries to engineering applications. This article aims to systematically review the research progress in MSMs. First, it will begin with the physical mechanisms of magneto‐X effects, providing an in‐depth analysis of the development and novel concepts of MR, magnetostrictive, giant magnetoelastic, magnetothermal, magnetically driven deformation, and magnetoelectric effects, along with their cutting‐edge applications (Section 2). Subsequently, based on these effects, the article will focus on reviewing breakthrough achievements in areas such as soft robotics and actuators, biomedicine (e.g., magnetically controlled drug delivery, hyperthermia therapy, and soft tissue engineering), flexible electronics (e.g., sensors, communication, and self‐powered devices), and energy and environment (e.g., energy harvesting and electromagnetic wave‐absorbing materials), while also analyzing the current key challenges and bottlenecks (Section 3). Finally, the article will outline future development opportunities and unresolved critical issues in the field (Section 4), emphasizing the need for interdisciplinary collaboration to drive holistic breakthroughs in MSMs from theory to application.

## 2 Magneto‐X Effects in Magnetic Soft Materials and Research Progress

MSMs exhibit a variety of unique magneto‐responsive effects under external magnetic fields, which form the foundation of their functionality. Among the most notable are their magnetic‐field‐controlled deformation and rheological properties: under an external magnetic field, magnetic nanoparticles within the material rapidly align to form chain‐ or column‐like microstructures, leading to significant changes in macroscopic mechanical properties such as viscosity, stiffness, and elasticity—a phenomenon known as the MR effect.

Additionally, some MSMs demonstrate remarkable magnetostrictive effects (undergoing volume or shape changes under magnetic fields) and magnetothermal effects (experiencing temperature variations during magnetization/demagnetization processes). More importantly, in specific composite structures or multiferroic soft materials, effects such as magnetoresistance (changes in electrical resistivity with magnetic field variation) and magnetoelectric effects (coupling between magnetic and electric fields, enabling magnetic control of electric properties or electric control of magnetic properties) may also emerge. These multifunctional characteristics endow MSMs with unique application potential in fields such as intelligent sensing, reconfigurable structures, biomedical devices, and electronic components.

### 2.1 Magnetorheological Effect

Magnetorheological (MR) effect is one of the most prominent phenomena in MSMs, referring to the controllable and reversible changes in the material's rheological or mechanical properties (such as viscosity, Young modulus, and yield stress) under an external magnetic field. This effect is observed in fluids, gels, and elastomers.

In MR fluids, when a magnetic field is applied, the suspended magnetic particles rapidly align (within milliseconds) along the magnetic field lines and form anisotropic chain‐ or column‐like structures. The formation of these structures causes the material to transition from a liquid‐like state to a nearly solid state, with its apparent viscosity increasing by several orders of magnitude and generating significant yield stress. MR materials typically comprise several distinct systems, depending on the medium used, such as MR fluids, MR foams, MR gels, and MR elastomers [60]. MR fluids have gained widespread application due to their low operational noise, rapid on‐site response, relative insensitivity to minor contaminants or dust, and ease of control [61]. On the other hand, despite their notable advantages and numerous potential applications, MR fluids suffer from drawbacks including sealing issues caused by leakage of the carrier liquid, environmental contamination, and particle sedimentation [62]. These limitations restrict the further expansion of their engineering applications.

Magnetic hydrogels are composite materials formed by integrating magnetic particles into a hydrogel matrix (e.g., natural polymers like sodium alginate and chitosan, or synthetic polymers such as polyacrylamide and polyethylene glycol). Due to their high‐water content and excellent biocompatibility, magnetic hydrogels have garnered significant attention in the biomedical field, with applications such as drug delivery carriers or tissue engineering scaffolds. Magnetic elastomers are created by embedding magnetic particles within an elastic polymer matrix. These materials exhibit rubber‐like elasticity and are capable of undergoing large‐scale reversible deformations [63, 64].

MR fluids typically consist of micron‐sized soft magnetic particles (such as the most widely used magnetizable medium: carbonyl iron [63]) and additives [64] dispersed in an oil‐based or aqueous carrier. Their viscosity and yield stress undergo significant, reversible changes under the influence of a magnetic field. Aqueous‐based magnetic fluids are typically used in biomedical fields, such as magnetic resonance imaging contrast agents and magnetic targeting drug delivery systems [65, 66]. Oil‐based magnetic fluids are primarily applied in engineering domains, including sealing [67], lubrication [68, 69], damping and vibration absorption [70], and precision polishing [71]. The performance of MR fluids is primarily determined by factors such as the shape, size, and concentration of soft magnetic particles [72, 73]. Additives also significantly influence the properties of MR fluids. For instance, urea used as a pore‐forming agent affects the thickness of the Fe_3_O_4_ shell layer, and a reduced shell thickness notably enhances suspension stability and rheological performance [74]. Similarly, the addition of lecithin‐based worm‐like micelles in MR fluids results in improved stability and a lower sedimentation rate [75].

In practical applications, MR fluids are required to simultaneously possess high shear yield strength, low zero‐field viscosity, and good sedimentation stability. However, these properties are often mutually constraining and difficult to balance. Traditional monodisperse particle systems, such as those using only micron‐sized carbonyl iron powder, suffer from issues including large interparticle gaps, weak chain structures, and rapid sedimentation. While nanoparticles can fill these gaps, their incorporation leads to a sharp increase in viscosity and higher costs. Du et al. [70] developed a high‐performance bimodal MR fluid using hybrid FeCo nanoparticles and carbonyl iron particles. The material simultaneously achieved enhanced shear yield strength (58.3 kPa), improved sedimentation stability (82.6%), and maintained low zero‐field viscosity (1.25 Pa·s).Using DC arc plasma‐synthesized FeNi particles, the team created bimodal MR fluids with 65 wt.% particle content, demonstrating a new strategy for balanced MR fluids design with significant engineering potential [76].

**FIGURE 2:**
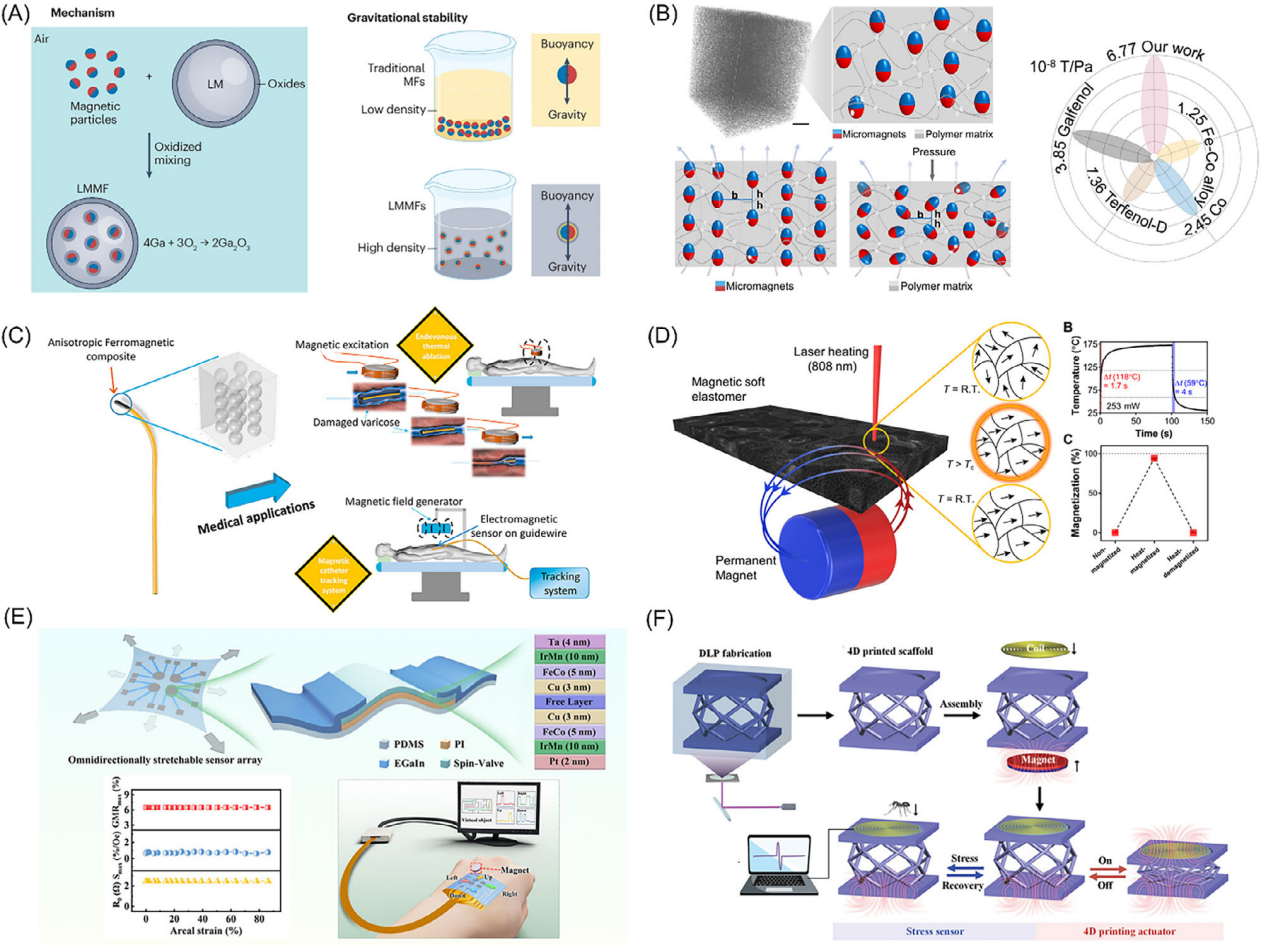
Research Progress in Magneto‐X Effects of Magnetic soft materials. (A) Suspension‐stable liquid metal magnetic fluid materials, Adapted with permission [45]. Copyright 2024, Nature Review Materials; (B) Giant magnetoelastic effect with a magneto‐mechanical coupling coefficient several times higher than that of rigid metal alloys, Adapted with permission [81]. Copyright 2021, Matter; (C) Flexible polymer composites based on micron‐sized iron oxide magnetic particles and their low‐frequency induction heating effect, Reproduced with permission [99]. Copyright 2021, Materials Today Chemistry; (D) Reprogrammable magnetic actuation technology based on laser‐induced phase transition, Reproduced with permission.[21] Copyright 2020, Science Advances; (E) Omnidirectionally stretchable spin‐valve sensor array based on the giant magnetoresistance effect, Adapted with permission [116]. Copyright 2025, ACS Nano; (F) Flexible magnetoelectric system based on electromagnetic induction of the magnetoelectric effect, Reproduced with permission.[129] Copyright 2024, Advanced Functional Materials.

Traditional magnetic fluid carriers (water, oil, or organic solvents) often suffer from low density and boiling points, limiting both the suspension stability and operational temperature range of magnetic fluids. In recent years, Professor Liu Jing's team developed liquid metal‐based magnetic fluid materials [45], as shown in Figure 2A, significantly transforming this research and application landscape. By introducing liquid metal as a carrier, they not only overcome these limitations but also endow the resulting liquid metal magnetic fluid with high electrical conductivity, substantially expanding the functionality of magnetic fluids. Moreover, due to the synergistic effects between the electrical conductivity of the liquid metal and the magnetism of the suspended particles, liquid metal‐based magnetic fluids exhibit complex multifunctional characteristics. To address the inherent non‐wettability between biocompatible particles (e.g., Fe_3_O_4_) and liquid metal (EGaIn), Shen et al. [77] developed a “reactive wetting” strategy. The team innovatively introduced silver nanoparticles as an intermediate layer, which react with indium at the interface to form Ag_x_In intermetallic compounds in situ. This newly formed reactive layer acts as a “wetting bridge,” enabling the originally incompatible Fe_3_O_4_ nanoparticles to be firmly anchored within the liquid metal matrix, thereby facilitating the fabrication of magnetic liquid metal robots that combine high magnetic stability with biocompatibility. After resolving the non‐wetting issue, another challenge emerged: how to suppress the alloying reaction between metallic magnetic particles (such as nickel, Ni) and gallium‐based liquid metals. This reaction causes the composite material to gradually solidify and lose fluidity. To address this, Huang et al. [78] proposed a core‐shell encapsulation strategy. The team synthesized Ni@SiO_2_ core‐shell nanoparticles as fillers, utilizing the chemically stable silicon dioxide (SiO_2_) shell as a physical barrier to effectively prevent direct contact between the inner nickel core and the external gallium‐based liquid metal. This design successfully suppressed the alloying reaction, resulting in a highly stable magnetic liquid metal paste, and demonstrated its application in magnetically recyclable, flexible self‐healing circuits.

To provide a clear comparison of their distinct characteristics, the key material systems, mechanisms, and properties of typical MR materials are summarized in Table 1.

**TABLE 1:** Comparison of major magnetorheological material systems.

Material type	Magnetic fluids	Magnetic hydrogels	Magnetic elastomers
Matrix/Carrier	Oil‐/Water‐based, Liquid Metal	Hydrogels (e.g., Alginate, PAM)	Rubber/Elastomers (e.g., PDMS)
Typical Magnetic Particles	Carbonyl Iron, FeCo/FeNi alloys, Fe_3_O_4_, Ni@SiO_2_	Fe_3_O_4_ nanoparticles	Micro‐/Nano‐sized ferromagnetic particles
Key Response Mechanism	Particle chain/column formation, drastic field‐induced increase in viscosity/yield stress	Magnetic response coupled with swollen polymer network	Particle‐matrix coupling, field‐induced Young modulus change & deformation
Main Advantages	Fast response (ms), high yield stress, excellent controllability, high electrical/thermal conductivity	High biocompatibility, tunable water content, injectable	No sedimentation, large reversible deformation, shape‐programmable
Limitations/Challenges	Particle sedimentation, sealing leakage, stability issues, complex fabrication (wettability issue)	Generally low mechanical strength, long‐term stability	Relatively low actuation stress, possible damping loss at high frequency
Representative Applications	Dampers, clutches, precision polishing, reconfigurable circuits, magnetic soft robots	Drug delivery, tissue engineering scaffolds	Soft robotics, tunable stiffness devices
Key References	[45, 60, 61, 63, 64, 70, 76, 77, 78]	[63, 64, 65, 66]	[63, 64, 79, 80]

### 2.2 Magnetoelastic, Giant Magnetoelastic, and Porous Coupled Systems

The magnetoelastic effect refers to the reciprocal coupling between a material's magnetic state and its mechanical deformation. While traditional research primarily focused on rigid alloys —where magnetic domain rotation induces subtle strains and magnetostriction is often treated as a defect to be minimized— the field has recently pivoted toward soft matter systems. Ongoing research continues to explore magnetostriction in bulk materials, even as the focus shifts toward systems characterized by large deformations, nonlinear coupling, and high tunability.

#### 2.2.1 The Giant Magnetoelastic Effect in Dense Elastomers

In 2021, Chen's group reported the “Giant Magnetoelastic Effect” in soft systems composed of micron‐sized magnets embedded in a polymer matrix [49, 81]. Unlike traditional materials, this effect utilizes a responsive magnetic network that generates significant changes in the surrounding magnetic field under biomechanical‐scale stresses (e.g., human pulse or respiration) without requiring an external field [82, 83]. The reported magneto‐mechanical coupling factor (6.77 × 10^−^^8^ T/Pa) is significantly higher than that of rigid counterparts [81], as shown in Figure 2B, enabling self‐powered sensing platforms [84]. In terms of theoretical modeling for the giant magnetoelastic effect, the team proposed an energy‐based homogenized hyperelastic model [85], integrated with Maxwell's equations, to predict changes in magnetic flux density within soft materials during deformation.

#### 2.2.2 Magnetoelastic Foams and Architected Porous Systems

Beyond dense elastomers, the research led by Kostas Danas and colleagues has fundamentally expanded the understanding of magnetoelastic foams and porous systems [86]. These porous architectures allow for substantial volumetric changes. Lin et al. [87]. demonstrated that in hard‐magnetic soft foams, the particle volume fraction evolves as a function of porosity during deformation. This dynamic microstructural evolution allows for robust and measurable magnetic flux changes that can be used to quantitatively infer complex loading scenarios, such as combined compression and shear, making them ideal for high‐sensitivity haptic sensing. Furthermore, Dorfmann and Ogden [88] provided a rigorous thermodynamic framework for these materials, addressing inconsistencies in Zeeman energy treatments to ensure the symmetry of the total Cauchy stress tensor under finite deformations.

#### 2.2.3 Nonlinear Anisotropy and Viscous Mechanisms in Ultra‐Soft Systems

Recent technical advancements have focused on “ultra‐soft” structures (Young modulus <10 kPa) where microstructural rearrangements play a dominant role. Perez‐Garcia et al. [89]. identified that residual anisotropies arising from magnetization lead to significant mechanical anisotropy even in the absence of external fields. This nonlinear behavior is highly dependent on the matrix stiffness and particle volume fraction, necessitating new topology optimization strategies for soft actuators. Additionally, Gonzalez‐Saiz et al. [90]. elucidated magnetic‐driven viscous mechanisms, showing that magnetic actuation can increase relaxation times by an order of magnitude. This effect enables “mechanical memory” and reprogrammable actuation protocols, where the material's yielding behavior can be tuned via magnetic stimuli history.

#### 2.2.4 Comparative Summary of Technical Approaches

Technically, the “Giant Magnetoelastic Effect” in dense systems excels in energy harvesting efficiency under low‐force inputs. In contrast, architected porous systems (Danas group) and ultra‐soft composites (Garcia‐Gonzalez group) offer superior tunability for complex shape‐morphing and quantitative sensing by exploiting volumetric compressibility and history‐dependent viscoelasticity. This shift from descriptive phenomenology to microstructurally‐informed constitutive modeling represents the current frontier in magneto‐active material research.

The distinct features, mechanisms, and application potentials of the three main categories of magnetoelastic systems discussed above are juxtaposed in Table 2 for a structured comparison.

**TABLE 2:** Comparison of magnetoelastic systems and their characteristics.

System category	Giant magnetoelastic (dense) elastomers	Magnetoelastic foams / architected porous systems	Magnetic ultra‐soft composites
Core Structural Feature	High density of micro‐magnets embedded in elastomer	Compressible porous architecture with tunable porosity	Ultra‐low Young modulus matrix (<10 kPa), low filler fraction
Key Magneto‐Mechanical Coupling Mechanism	Deformation of magnetic network induces large local magnetic field change, generating highly sensitive passive magnetic signal	Deformation alters particle volume fraction and distribution, leading to measurable magnetic flux change	Residual magnetic anisotropy (from magnetization) dictates mechanical anisotropy; pronounced viscous mechanisms
Performance Merit	Extremely high magneto‐mechanical coupling coefficient, suitable for micro‐force/energy harvesting	Large volumetric strain, high sensitivity, ability to decouple complex loading	Excellent shape‐morphing capability, mechanical memory, and reprogrammability
Primary Application Direction	Self‐powered sensing, low‐power physiological monitoring	Haptic sensing, shape morphing, pressure mapping	Soft actuators, programmable metamaterials
Key Challenge	Small absolute signal strength, requires sensitive detection	Complex constitutive modeling, fatigue life assessment	Strong mechanical nonlinearity, complex design and control
Representative References	[49, 81, 82, 83, 84, 85]	[86, 87, 88]	[89, 90]

### 2.3 Magnetothermal and Magnetocaloric Effects

The magnetocaloric effect (MCE) is a magnetothermodynamic phenomenon where certain materials undergo temperature changes due to variations in magnetic entropy Δ*S_m_* under an external magnetic field [91]. While MCE is the thermodynamic foundation for magnetic refrigeration, the broader magnetothermal effect—referring to heat generation via magnetic loss mechanisms (such as Néel/Brownian relaxation or hysteresis loss) under an alternating magnetic field (AMF)—is the driving force behind magnetic hyperthermia therapy. Magnetic nanoparticle‐based magnetic hyperthermia(MNPs‐MH) is a promising novel approach for treating solid tumors, MSMs containing nanoparticles is injected into tumor tissues. Under an AMF, these particles convert magnetic energy into thermal energy, raising the tissue temperature above 42°C to destroy cancer cells [92]. The most commonly used materials for this therapy are iron oxide nanoparticles in the size range of 10–100 nm, particularly magnetite (Fe_3_O_4_) or maghemite (γ‐Fe_2_O_3_). These materials are preferred due to their lower toxicity compared to other magnetic materials, tunable magnetic properties, relatively simple synthesis, and good stability [93]. Strategies to enhance the antitumor efficacy of MNPs‐MH therapy typically include: (1) Optimizing the size, composition, morphology, and surface properties of magnetic nanoparticles to achieve sufficient and effective heating characteristics; (2) Gaining deeper insights into the impact of localized inductive heating on the disruption of cellular/subcellular structures, thereby improving the therapeutic outcomes of MNPs‐MH in antitumor treatments. For instance, Chen et al. [94] developed an injectable reactive hydrogel capable of inducing mutually enhanced mild magnetic hyperthermia and ferroptosis to improve the antitumor efficacy of MNPs‐MH. This work not only provides a new strategy for the clinical translation of MNPs‐MH but also confirms the synergistic antitumor mechanism between MNPs‐MH and ferroptosis. Zhang et al. [95] successfully developed a magnetic protein nanocage with ultrahigh magnetothermal efficiency through genetic engineering and biomimetic mineralization strategies. Each nanocage can load approximately 31 590 iron atoms, significantly surpassing the capacity of ferritin (fMIONs, ∼3394 atoms). This structure achieves an ultrahigh magnetothermal conversion efficiency, with a specific absorption rate value as high as 2390 W/g.

Furthermore, induction heating effects in micron‐sized magnetic particles have been exploited for macro‐scale applications. In recent studies on micron‐sized magnetic particles, Xiang et al. [96, 97] utilized the low‐frequency induction heating effect to fabricate flexible polymer composites containing micron‐sized iron oxide magnetic particles via 3D printing technology, exploring their potential in medical applications, particularly for the thermal ablation treatment of varicose veins. The study found that inducing ordered alignment of magnetic particles in the polymer through an external magnetic field to achieve magnetically anisotropic composites can significantly enhance the magnetothermal conversion efficiency along the direction of particle alignment [98, 99], as shown in Figure 2C.

The reversible process of the MCEforms the basis of magnetic refrigeration. Compared to traditional gas‐compression refrigeration technologies, magnetic refrigeration is considered more energy‐efficient and environmentally friendly. However, current magnetocaloric materials predominantly rely on rare‐earth elements, which are costly and resource‐scarce. Consequently, the development of high‐performance, rare‐earth‐free magnetocaloric materials has become a key research focus in recent years. Levinsky et al. [100] synthesized a novel layered coordination polymer, Co_4_(OH)_6_(SO_4_)_2_[enH_2_], featuring a brucite‐type structure with interlayers separated by ethylenediammonium ions. This material exhibits a significant MCEnear liquid hydrogen temperatures (∼20 K) under a magnetic field of just 1–2 T, making it suitable for permanent magnet‐driven and highly efficient magnetic refrigeration in the liquid hydrogen temperature range. When developing novel magnetocaloric materials, the traditional experimental‐driven discovery process is slow and costly. To address this, J. Court et al. [101] established, for the first time, an end‐to‐end inverse design pipeline that spans from literature mining to material generation, significantly accelerating the discovery of magnetic materials. The automatically constructed database achieved an accuracy of 86%, covering multiple compounds and properties. The generated materials showed high structural consistency with DFT‐optimized results (with an average bond length variation of only 1.9%). The predicted candidate materials performed comparably or even superior to known materials in terms of Curie temperature, magnetic entropy change, and relative cooling power.

### 2.4 Magneto‐Driven Deformation Effect

The magneto‐driven deformation effect is the cornerstone mechanism enabling the multifunctionality of MSMs. It refers to the use of external magnetic fields to induce complex, large‐scale, and reversible shape changes in materials. The key to achieving such programmable and time‐dependent deformation modes lies in the pre‐designed, non‐uniform magnetization distribution patterns embedded within the elastomeric matrix.

The rational design and predictive modeling of these materials must be grounded in a rigorous continuum mechanics framework that accounts for the severe nonlinearities of magneto‐elastic coupling. Dorfmann and Ogden [88] recently clarified essential energy considerations in the finite deformation context, demonstrating that a self‐consistent energy formulation is required to ensure the symmetry of the Cauchy stress tensor—a critical theoretical detail often overlooked in earlier models that relied solely on Zeeman magnetic energy. Building upon these foundations, Stewart and Anand [102] introduced a comprehensive magneto‐viscoelastic theory, providing the mathematical tools to capture the path‐dependent and time‐sensitive behaviors of hard‐magnetic elastomers. Their framework is particularly effective in modeling dynamic phenomena, such as the snap‐through instability of bistable arches. Furthermore, to bridge the gap between microstructural features and macroscopic response, Narayanan et al. [9]. developed a micromechanics‐based constitutive model. By accounting for particle‐matrix interactions and nonlinearities, this model effectively predicts magneto‐mechanical hysteresis, offering a refined perspective on material‐level control.

Beyond constitutive modeling, the interplay between magnetic fields and structural mechanics offers unique opportunities for functional design. Pedro M. Reis and co‐authors [103, 104] have made fundamental contributions by revealing that magnetization in incompressible hard‐magnetic elastomers remains stretch‐independent during finite deformations—a finding that refines the energetic description of these materials. Moreover, they demonstrated that magnetic actuation can dynamically tune the buckling strength of elastic shells, transforming traditional structural instabilities into controllable mechanisms for multimodal motion and programmable logic.

In terms of engineering implementation, while the Xuanhe Zhao research team has significantly advanced the field through 3D printing and topology optimization of ferromagnetic domains [79, 80], the design of ultra‐soft systems (stiffness < 10 kPa) requires addressing fundamental challenges such as nonconvexity and the loss of ellipticity. To this end, Perez‐Garcia et al. [89]. proposed an advanced physics‐constrained optimization framework. Their work leverages residual mechanical anisotropies—microstructural rearrangements induced by residual magnetization—to achieve sophisticated shape‐morphing capabilities that remain stable under complex loading paths, offering a more robust alternative to conventional optimization strategies.

Current magnetic programming in soft matter relies on templating [105, 106] or 3D printing [79], which permanently locks the magnetic field during fabrication. This fixed distribution, combined with slow manufacturing speeds, limits mass production and practical utility of magnetically driven soft robots. Researchers are therefore developing reprogrammable magnetic actuation technologies. For example, Qi et al. [107] created a magnetoactive soft material with reconfigurable shape‐morphing and self‐sensing capabilities. Using a thermal‐assisted magnetic programming strategy, local heating and magnetic fields enable repeated reprogramming of magnetic particle chains for flexible macroscopic shape control. Alpan et al. [21] demonstrated a method to remagnetize soft robots by heating embedded permanent magnetic particles above their Curie point. During cooling, an external magnetic field realigns the magnetic domains, allowing redistribution of the internal magnetic pattern, as shown in Figure 2D. Sun et al. [108] used materials sensitive to magnetic resonance and low‐temperature phase transitions. High‐frequency magnetic fields selectively heat specific robot regions, enabling rapid in‐situ reprogramming of single or multiple robots. Liu et al. [109] took a non‐thermal approach: by utilizing two magnetic particle types with distinct coercivities, an external field selectively reorients magnetization in different parts of a micro‐device, enabling reusable programming.

The non‐local and nonlinear nature of magnetically driven deformation poses challenges for modeling and simulation, hindering theoretically driven rational design. Existing methods, such as the finite element method, suffer from high computational costs when dealing with sparsely distributed magnets and heterogeneous media, and struggle to handle large deformations. To address the challenge of modeling magnetically driven deformation, Mai et al. [110] proposed a 2D model based on beam theory, though it is only applicable to planar structures. In contrast, the Alkuino team developed a versatile open‐source framework based on Lattice Spring and Point‐Dipole Models [111], successfully simulating various nonlinear mechanical phenomena such as bistable behavior, magnetic response of shape‐memory polymers, gradient magnetic fields, and phase transitions in magnetic metamaterials. This framework supports extensions to 3D simulations, multi‐physics coupling, and collective behavior studies, providing an efficient tool for the design of intelligent materials.

A summary of the predominant magnetic programming strategies and theoretical modeling approaches, highlighting their respective principles and trade‐offs, is presented in Table 3.

**TABLE 3:** Comparison of magnetic programming strategies and modeling approaches for magnetically driven deformation.

Category	Method/strategy	Core principle	Advantages	Limitations	Key references
Magnetization Programming	Template‐Assisted Magnetization	Curing material near hard‐magnetic templates to imprint specific magnetization patterns	Simple, capable of complex patterns	Pattern is fixed, not reprogrammable	[105, 106]
	3D Printing Magnetization	Applying magnetic field in situ during printing to write magnetization direction	High spatial resolution, high design freedom	Requires specialized setup, slow printing speed	[79, 80]
	Thermal‐Assisted Reprogramming	Heating above Curie point to reset magnetization, applying new field during cooling	Re‐programmable, suitable for various materials	Requires thermal management, limited cycles	[21, 107]
	Selective Joule‐Heating Reprogramming	Using high‐frequency AMF to selectively heat local regions for re‐magnetization	Fast, in situ, selective reprogramming	Requires materials with high magnetothermal response	[108]
	Dual‐Component (Heterogeneous) Magnetization	Using particles with different coercivities for selective reorientation of magnetization	No heating needed, room‐temperature operation	Complex material synthesis and formulation	[109]
Modeling & Simulation	Continuum Mechanics Framework	Finite deformation magneto‐visco‐hyperelastic theory	Physically rigorous, suitable for constitutive analysis	High computational cost, difficult for complex microstructures	[88, 102]
	Lattice Spring—Point Dipole Model	Discrete method combining near‐field mechanics and far‐field magnetic interaction	Computationally efficient, handles large deformation and complex structures	Limited resolution of some continuum details	[111]
	Micromechanics‐Based Model	Predicts macroscopic hysteresis from particle‐matrix interactions	Bridges micro‐mechanisms and macro‐performance	Many model parameters, complex calibration	[9]

### 2.5 Magnetoresistance Effect and Magnetoelectric Effect

The magnetoresistance effect refers to the physical phenomenon where the electrical resistance of a material changes under an applied magnetic field. Common types of magnetoresistance include giant magnetoresistance, anisotropic magnetoresistance, and tunneling magnetoresistance. The giant magnetoresistance effect is a key phenomenon in spintronics, commonly observed in multilayer structures with alternating magnetic and non‐magnetic layers. Its resistance decreases significantly as the external magnetic field increases. Although initially discovered in rigid metals, the underlying mechanism has been successfully applied to flexible electronics [112]. At the microscopic level, the electron scattering probability is modulated by the relative orientation between electron spin and the magnetization direction of the medium, enabling resistance control.

#### 2.5.1 Giant Magnetoresistance (GMR)

In the field of magnetic sensors, conventional GMR sensors based on rigid substrates like silicon struggle to withstand tensile strain. To overcome this limitation, researchers have developed multiple flexible technology pathways. For instance, Melzer et al. [113] utilized thermal expansion coefficient differences to induce wrinkled structures on PDMS, increasing the device stretchability to 4.5% while maintaining over 50% GMR ratio. Zhang et al. [114] proposed an electrochemical exfoliation method for rapid and damage‐free transfer of ultrathin GMR devices, significantly improving manufacturing efficiency.

Against this background, the Run‐Wei Li team has systematically advanced the performance boundaries of stretchable GMR sensors. Their early work, using wrinkled and ribbon‐integrated structures, enabled the sensors to maintain approximately 9.9% GMR ratio under 25% strain [115]. More recently, they further developed a strategy combining a “Young modulus distribution structure” with liquid metal electrodes [116], successfully constructing an omnidirectionally stretchable spin‐valve sensor array, as shown in Figure 2E. This device maintains a stable GMR ratio of about 8% under extreme omnidirectional strain up to 86%, with a sensitivity of 0.93%/Oe and durability over thousands of cycles. Ultimately, it enables contactless interactive e‐skin that conforms to the skin and operates reliably under complex deformations, demonstrating broad application potential.

In the field of electronic skin and biosensors, Dr. Denys Makarov's team presents a novel flexible magnetoreceptive electronic skin based on the giant GMR effect and electrical resistance tomography. The technology enables continuous, large‐area (120×120 mm^2^), high‐resolution (<1 mm), and low‐power magnetic field sensing using a single continuous sensing layer and a minimal number of electrodes, while also offering transparency, flexibility, and breathability. It provides an innovative solution for applications such as undisturbed extended reality interaction and hygienic contact lens interfaces [117]. Meanwhile, Makarov's team has also researched and developed a transparent magnetoresistive sensor. By employing a magnetic field‐guided printing process and a locally entangled nanowire structure, this sensor achieves a high transparency of approximately 85% while endowing it with excellent mechanical flexibility and stretchability, offering a new strategy for fabricating high‐performance transparent flexible magnetic sensors [118]. Su et al. developed a “lab‐on‐a‐needle” platform based on ultra‐flexible GMR sensors [119]. Its performance—with a magnetoresistance ratio of 5.2% and sensitivity of 0.13%/Oe—is comparable to that of rigid devices, and it remains stable after 500 deformation cycles. The platform has been successfully applied to real‐time detection of canine osteosarcoma cells. Separately, Stanford University researchers created a GMR‐based sensor for early diagnosis of hepatocellular carcinoma [120], capable of automatically detecting biomarkers. Controlled via a smartphone application, this low‐cost, portable device is suitable for point‐of‐care testing in resource‐limited areas. These advances demonstrate that the stringent requirements for detection performance and portability in medical diagnostics are driving the commercialization of flexible magnetoresistive technology.

Ota et al. has developed a novel strain sensor based on the GMR effect [121], achieving for the first time simultaneous detection of both strain magnitude and direction. The sensor features a cobalt‐based ferromagnetic/non‐magnetic/ferromagnetic trilayer structure, whose resistance varies with the relative angle between the magnetization directions of the two ferromagnetic layers. Innovatively, using only cobalt material, the team leveraged the magnetoelastic effect to regulate the magnetic anisotropy of the sensitive layer, enabling the magnetization direction to rotate with the strain direction.

#### 2.5.2 Anisotropic Magnetoresistance (AMR)

The anisotropic magnetoresistance effect refers to the physical phenomenon where the electrical resistance of a magnetic material changes with the angle between its internal magnetization direction and the current direction. This characteristic makes magnetic materials an ideal choice for highly sensitive sensors. In recent years, combining the AMR effect with the mechanical flexibility of magnetoelastic materials has given rise to a highly promising new application direction: flexible magnetoelastic sensors. The core mechanism lies in the fact that when a flexible magnetoelastic material undergoes mechanical strain, the magnetoelastic anisotropy within the material changes, causing its magnetic domain structure and overall magnetization direction to shift. This “force”‐induced change in magnetic state is then precisely read out in the form of an “electrical” signal through the AMR effect, thereby achieving direct conversion from mechanical deformation to electrical signals.

At macroscopic or micron scales, the observed behavior represents a statistical average of numerous magnetic domains, which obscures the most fundamental physical processes of stress‐mediated magnetic regulation. To fundamentally understand this mechanism, it is essential to directly reveal the physical process of mechanical stress modulating magnetic domains at the nanoscale. To achieve this, Kong et al. [122] employed advanced in‐situ Lorentz transmission electron microscopy and electron holography to directly observe the dynamic evolution of magnetic domains in ferromagnetic nickel thin films under tensile strain for the first time. The core innovation of this work lies in providing direct visual evidence of strain‐induced “magnetic hardening”: as strain increases, periodic 180° magnetic domain walls perpendicular to the stress axis form within the material. This magnetization transition process is reversible and can be regulated through nanoscale structural dimensions, thereby revealing the physical origin of the magnetoelastic effect at the most fundamental level.

After directly observing strain‐induced changes in magnetic domains, the next step is to quantify the complex competition between mechanical stress and intrinsic magnetic properties. In their study, Patel et al. [123] thoroughly investigated the interaction between magnetocrystalline anisotropy and magnetoelastic anisotropy in epitaxial cobalt films. While the team initially aimed to create a simple in‐plane uniaxial anisotropic system, ferromagnetic resonance measurements revealed significantly more complex magnetic behavior. Their key contribution lies in demonstrating that this complexity stems from an energy competition between the material's inherent magnetocrystalline anisotropy and the strain‐induced magnetoelastic anisotropy introduced by the substrate. Through precise quantitative analysis, the study successfully separated and evaluated the contributions of these two competing anisotropies, providing a critical physical model for understanding and controlling the macroscopic magnetic response of strained thin films.

Applying the fundamental magnetoelastic effect to functional devices, particularly in flexible electronics, is key to transforming this physical phenomenon into practical technology. A recent study by Su et al. [124] demonstrates such an application: they successfully fabricated epitaxially strained cobalt‐iron films on flexible mica substrates and systematically investigated their anisotropic magnetoresistance. The innovation of this work lies in directly correlating the magnetoelastic effect with electrical transport properties—when the flexible mica substrate is bent, the applied mechanical strain induces strong uniaxial magnetic anisotropy in the CoFe film through the magnetoelastic effect. This strain‐induced magnetic anisotropy subsequently modulates the material's AMR response. The results show that simply bending the substrate can effectively alter the device's magnetoresistance curve, proving the feasibility of regulating magnetic and electrical properties through mechanical strain and laying the foundation for novel flexible magnetoelastic sensors.

#### 2.5.3 Tunneling Magnetoresistance (TMR)

The tunneling magnetoresistance effect refers to the phenomenon in a magnetic tunnel junction where the tunneling current changes significantly with the relative orientation of the magnetization directions in the two ferromagnetic layers. By combining it with the magnetoelastic effect of ferromagnetic materials, a highly promising sensing application is formed: when the material is subjected to mechanical stress or strain, its internal magnetic anisotropy changes, leading to a shift in the magnetization direction. This “force”‐induced change in magnetic state can be sensitively “read” as a resistance signal by the TMR structure, enabling precise measurement of minute stress or deformation.

Utilizing highly sensitive TMR sensors for magnetoelastic effect detection is crucial for achieving high‐precision stress sensing. In a groundbreaking study, Dewa et al. [125] employed a linear triaxial TMR sensor to non‐invasively measure minute changes in the material's self‐magnetic flux density induced by mechanical stress—a direct manifestation of the magnetoelastic effect. This approach enabled grain‐level high‐resolution imaging of stress distribution at fatigue crack tips. The team successfully mapped stress concentration zones near cracks of varying depths and angles, effectively transforming invisible mechanical stress fields into measurable magnetic field distributions. This research demonstrates the application of TMR sensors as high‐sensitivity read heads, offering significant value for non‐destructive testing and structural health monitoring.

#### 2.5.4 Magnetoelectric Effect

The magnetoelectric effect refers to the phenomenon where a material develops electrical polarization under an external magnetic field or becomes magnetized under an external electric field. This effect originates from both intrinsic effects observed in single‐phase materials and extrinsic effects generated in composite materials. Intrinsic magnetoelectric coupling occurs in multiferroic materials, which require simultaneous ferroelectricity and ferromagnetism [126]. However, strong magnetoelectric coupling in single‐phase materials is rare and typically observable only at temperatures well below room temperature, limiting their practical applicability. Composite material systems achieve strong magnetoelectric coupling by mechanically integrating two distinct materials [127]. A flexible magnetoelectric system based on magnetostrictive and piezoelectric effects is formed by combining magnetostrictive materials with piezoelectric materials. When a magnetic field is applied to the composite, the magnetostrictive layer alters its shape and dimensions. The mechanical strain is then transferred through the interface to the adjacent piezoelectric layer, generating a voltage via the piezoelectric effect, thereby completing the magneto‐electric energy conversion. Recently, Su et al. [128] proposed a flexible magnetoelectric system based on electromagnetic induction, which disperses low‐elastic‐Young modulus magnetic material and conductive copper coils within a flexible medium. This design enables synergistic interaction between the magnetic and conductive components: when the elastomer is compressed or recovers, the distance between the magnets and coils changes, altering the magnetic flux and generating an induced electromotive force. Furthermore, the team integrated electromagnetic actuation and induction mechanisms into a 4D‐printed structure [129], creating an electromagnetic architecture capable of ultrafast deformation and ultrahigh sensitivity sensing. By ingeniously integrating and coupling different physical effects in composite materials, multifunctionality unattainable with traditional single materials can be achieved, thereby expanding their application boundaries.

Table 4 provides a systematic comparison of the fundamental characteristics, performance metrics, and application prospects of different magnetoresistive and magnetoelectric effects in the context of flexible sensing.

**TABLE 4:** Comparison of magnetoresistive and magnetoelectric effects for flexible sensing applications.

Effect type	Giant magnetoresistance (GMR)	Anisotropic magnetoresistance (AMR)	Tunneling magnetoresistance (TMR)	Magnetoelectric (ME) effect
Physical Basis	Spin‐dependent scattering	Electron scattering anisotropy	Spin‐polarized tunneling	Strain‐mediated coupling / Electromagnetic Induction
Typical Materials/Structures	[Co/Cu/Co] multilayers, spin valves	Ni, NiFe, CoFe thin films	CoFeB/MgO/CoFeB magnetic tunnel junctions	PVDF/FeGa, PZT/Terfenol‐D composites; Coil‐Magnet‐Elastomer systems
Key Performance Metrics (for Flexible Devices)	GMR Ratio (8%–12%), Sensitivity (%/Oe), Stretchability (up to >80%)	AMR Ratio (1%–5%), Magnetoelastic coupling coefficient	TMR Ratio (>100% at RT), extremely high sensitivity	ME Voltage Coefficient (mV/cm Oe), Sensitivity, Bandwidth
Advantages (in Flexible Sensing)	High sensitivity, fast response, low power consumption	Simple structure, sensitive to field direction	Highest sensitivity, strong signal at low field	Direct magnetic‐to‐electric conversion, self‐powering capability
Challenges	Performance retention under large strain, CMOS integration	Relatively small signal, temperature sensitivity	Complex fabrication, integration into flexible systems	Interface optimization in composites, trade‐off between low‐frequency performance and sensitivity
Application Examples	E‐skin, biosensors, non‐contact interfaces	Angle/position sensors, stress/strain sensors (coupled with magnetoelastic layer)	High‐resolution stress imaging, bio‐magnetic detection, NDT	Energy harvesters, low‐power magnetic field sensors, multifunctional systems
Key References	[113, 114, 115, 116, 119, 120, 121]	[122, 123, 124]	[125]	[126, 127, 128, 129]

## 3 Multidisciplinary Research Advances in Magnetic Soft Materials

MSMs enables breakthrough applications through remote, non‐contact magnetic control. Programmable soft microrobots perform targeted drug delivery and minimally invasive surgery in biomedicine, while magnetostrictive/rheological effects power high‐sensitivity sensors and adaptive dampers. Optimized magnetic properties support high‐frequency components for next‐generation power and data storage. Through material‐structure‐programming synergy, these materials are advancing soft robotics, wearables, and medical technologies with transformative potential.

### 3.1 Soft Robotics and Actuators

MSMs shows great potential in the field of soft robotics and actuators. Compared to traditional rigid robots, soft robots possess significant advantages due to their flexibility, compliance, and adaptability to complex environments. MSMs enable non‐contact, multi‐degree‐of‐freedom wireless control, making them particularly suitable for developing untethered and programmable deformable soft machines. These magnetically responsive materials can undergo various precise and complex deformations when driven by external magnetic forces or torques, as their programmed magnetic dipoles form magnetic domains with specific configurations. In recent years, researchers have made remarkable progress through material innovation and structural design, focusing on key directions such as enhancing drive performance, simplifying motion control, and integrating multifunctional sensing.

**FIGURE 3:**
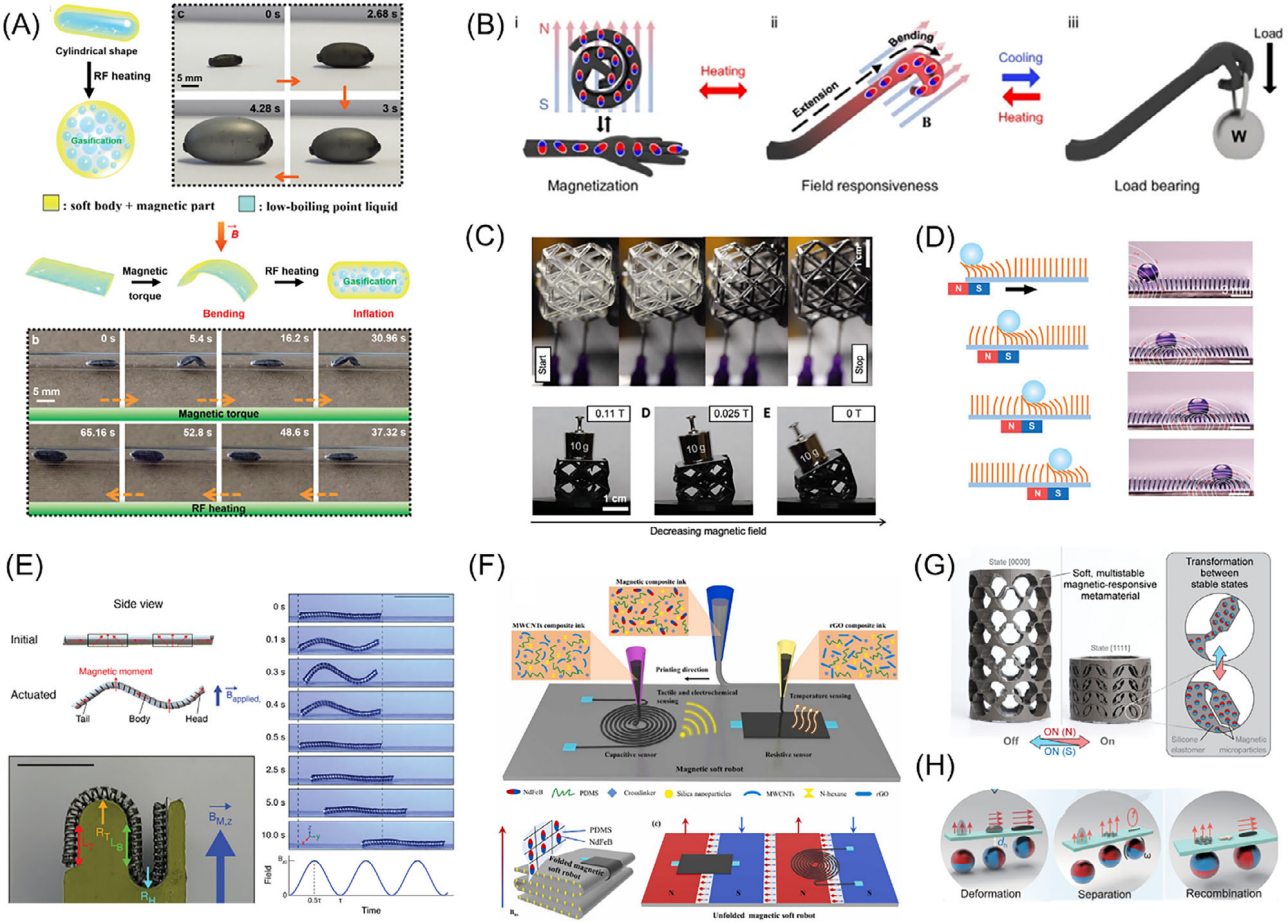
Flexible Robots and Actuators Based on Magnetic soft materials. (A) Wireless miniature magnetic phase‐transition soft actuator combining magnetic drive and pneumatic expansion, Adapted with permission [130].Copyright 2022, Advanced Materials; (B) Multifunctional magnetic actuator with rigidity‐flexibility switching ratio exceeding 2700×, based on phase‐change shape‐memory polymers and neodymium iron boron particles, Reproduced with permission [19]. Copyright 2024, Nature Communications; (C) Magnetic‐field‐regulated mechanical metamaterial with wide dynamic range and fast reversible response, utilizing MR fluid, Adapted with permission.[48] Copyright 2018, Science Advances; (D) Magnetically driven droplet manipulator based on magnetic micropillar array, Adapted with permission.[131] Copyright 2022, Advanced Functional Materials; (E) Three‐dimensional helical soft robot capable of multi‐actuator directional control under a single magnetic field, Adapted with permission.[132] Copyright 2023, Advanced Materials; (F) Multi‐dimensional deformable magnetic‐driven robot fabricated via multi‐material integrated printing, Reproduced with permission.[133] Copyright 2023, Additive Manufacturing; (G) Magnetically driven soft robot maintaining multiple stable configurations without continuous energy input, Reproduced with permission.[134] Copyright 2025, Science Advances; (H) Scale‐reconfigurable miniature ferrofluid robot, Adapted with permission.[135]Copyright 2022, Science Advances.

A core challenge lies in overcoming the inherent “strength‐flexibility” trade‐off in soft actuators to achieve powerful mechanical output. To address this issue, researchers have proposed various innovative strategies. For example, Tang et al. [130] ingeniously combined magnetic actuation with pneumatic expansion, using radiofrequency fields to heat an internal low‐boiling‐point liquid, causing it to vaporize and generate substantial internal pressure, as shown in Figure 3A. This approach increased the actuator's output force and work capacity by several orders of magnitude, opening new possibilities for micro‐soft robots in demanding fields such as healthcare. Another approach involves introducing variable stiffness mechanisms. Seong et al. [19] integrated phase‐change shape‐memory polymers with ferromagnetic particles, utilizing remote laser heating to enable rapid switching between rigid and flexible states (Figure 3B). Their system achieved a stiffness switching ratio exceeding 2700‐fold, effectively resolving the conflict between load capacity and motion flexibility. Similarly, Jackson et al. [48] infused MR fluid into 3D‐printed microtubes to create mechanical metamaterials with real‐time, reversibly tunable mechanical properties, providing both theoretical and practical foundations for controllable soft robots and adaptive protective devices (Figure 3C).

While enhancing drive performance, achieving precise and efficient control of soft robots remains a key research focus. At the microscopic manipulation level, Jing et al. [131] developed a magnetic micropillar array platform that enables high‐precision three‐dimensional droplet manipulation, overcoming the limitations of traditional methods such as weak driving force and confinement to two‐dimensional planes (Figure 3D). For macroscopic motion control, Lee et al. [132] utilized a thermal stretching process to create dual‐component elastomer fibers with three‐dimensional helical structures (Figure 3E). This breakthrough allows simultaneous control of multiple robots moving in different directions under a single fixed magnetic field, significantly simplifying the control system.

Toward more advanced “intelligent” soft robots, the integration of actuation, sensing, and energy management into a unified system is essential. Wang et al. [133] employed multi‐material integrated 3D printing to seamlessly incorporate different functional composites—responsible for actuation, temperature sensing, tactile sensing, and electrochemical sensing—into a single robotic structure(Figure 3F). This system successfully demonstrated targeted drug delivery and real‐time monitoring in a simulated gastric environment, marking a significant step forward in transforming magnetic soft robots from mere actuators into intelligent systems capable of perceiving and responding to their environment. In terms of energy efficiency, Greenwood et al. [134] devised an ingenious structural design that allows robots to “lock” energy in the form of elastic potential within their architecture, enabling the maintenance of multiple stable configurations without continuous energy input (Figure 3G). This “set‐and‐hold” mechanism for shape maintenance is critically important for long‐term implantable medical devices and energy‐efficient soft robots.

Traditional magnetic microrobots with fixed dimensions are limited to operating in confined spaces matching their size, but Fan et al. [135] developed a scale‐reconfigurable miniature ferrofluid robot capable of moving, deforming, and restructuring freely across drastically varying spatial scales (Figure 3H). This magnetic droplet‐based robot can split or merge into different sizes under magnetic control: centimeter‐scale units dominated by gradient forces for dragging in large spaces, millimeter‐scale units influenced by both magnetic torque and gradient forces, and micrometer‐scale units governed primarily by torque for rotational motion. In a simulated multi‐scale vascular network, the robot successfully traversed centimeter channels, split into millimeter units to navigate curved pathways, further divided into micron‐scale units to enter microscopic circular mazes, and ultimately merged back to larger dimensions.

In summary, these cutting‐edge studies collectively advance the comprehensive capabilities of magnetic soft systems in manipulation precision, driving force, control efficiency, and environmental interaction through enhancements in output performance, motion control optimization, and integrated sensing. They provide diverse insights and solutions for future disruptive applications in healthcare, micro‐nano manipulation, and beyond.

### 3.2 Biomedical Engineering

MSMs, with its exceptional mechanical compliance, enables safe and efficient interaction with biological tissues, demonstrating broad application prospects in the field of biomedical engineering. Its typical applications include targeted magnetically controlled drug delivery systems, precise magnetic hyperthermia platforms, adaptively growing magnetic soft tissue engineering scaffolds, and cellular mechanical stimulation platforms capable of simulating physiological microenvironments.

#### 3.2.1 Magnetically Controlled Drug Delivery

By encapsulating drugs within MSMs such as hydrogels or microcapsules, external magnetic fields can be utilized to precisely guide the drugs to targeted sites within the body, enabling controlled release [136, 137]. This is particularly crucial for targeted therapies like cancer treatment. Recent research advances have primarily focused on addressing several core challenges: achieving precise navigation within deep tissues, enabling controlled functional operations at target sites, enhancing the ultimate therapeutic efficacy of drug delivery into cells, and optimizing the biocompatibility of carrier materials.

Achieving precise navigation and targeted accumulation is the fundamental prerequisite for magnetically controlled drug delivery. In this regard, Kim et al. [138] transformed functional particles into Janus‐type magnetic particles by sputtering a nickel‐gold film onto their surfaces. They successfully demonstrated the feasibility of in vivo magnetic navigation and accumulation by using external magnets to attract and aggregate these particles in a mouse vascular model, laying the foundation for delivering drug carriers to specified areas.

After achieving precise navigation, on‐demand drug release and multifunctional integration have become key challenges. The Sun team designed a soft valve system based on the competition between magnetic gradient forces and torque [139], using low‐frequency magnetic fields to control movement and high‐frequency fields to trigger localized release, successfully decoupling motion and operation. This technology has demonstrated targeted delivery, selective multi‐drug release, and photothermal synergistic therapy in animal experiments, showcasing the application potential of diagnostic‐therapeutic integrated capsule robots.

To enhance intracellular drug delivery efficiency, research focus is shifting from tissue targeting to cellular targeting. Ye et al. [140] fabricated helical microrobots via two‐photon 3D printing and functionalized them with magnetic metal‐organic framework nanoparticles. This design integrates magnetic navigation with biological targeting, utilizing porous structures to load targeting molecules to break through the limitations of traditional passive diffusion, significantly improving the precision and efficacy of intracellular drug delivery.

**FIGURE 4:**
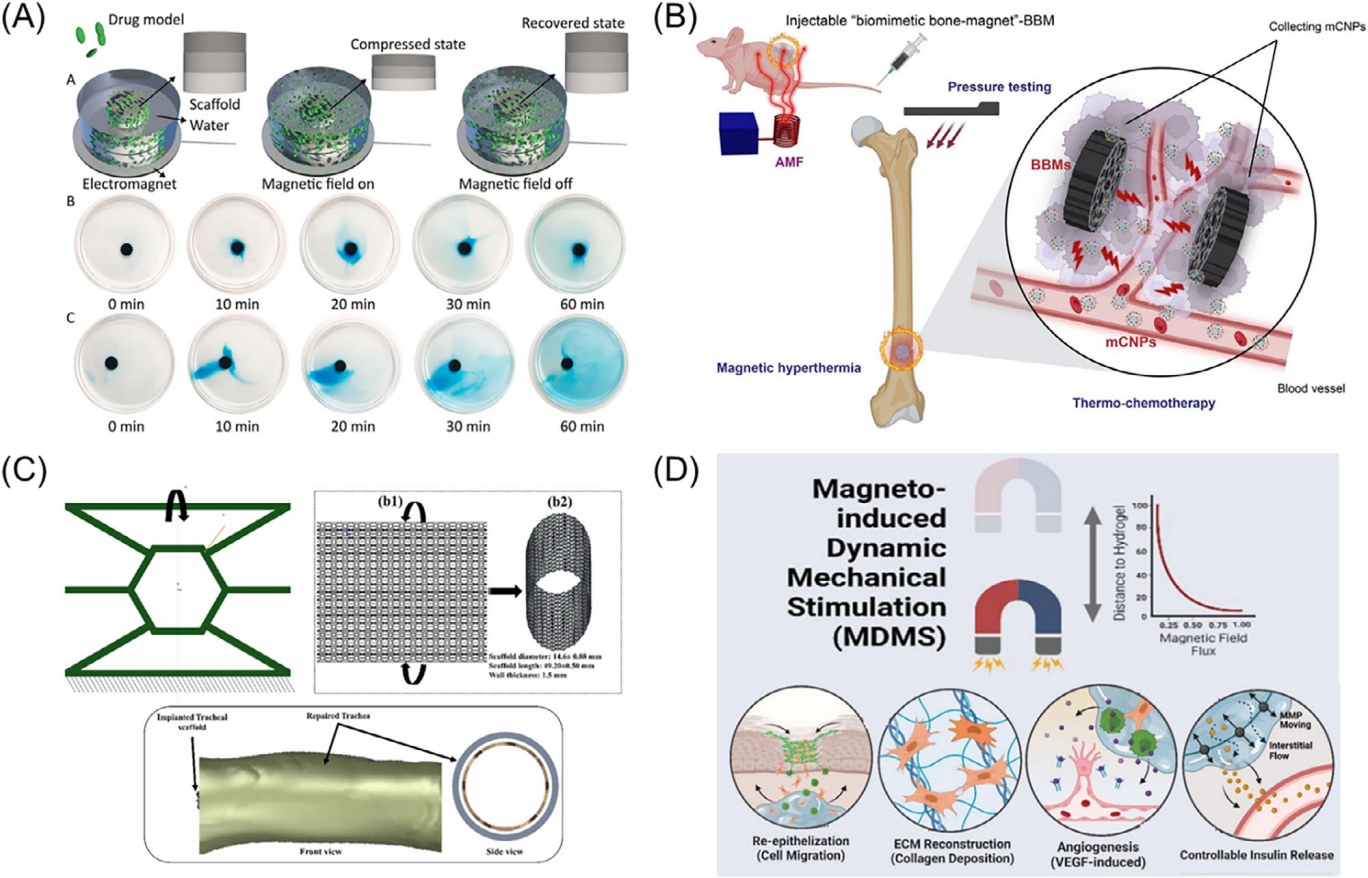
Bioengineering applications based on magnetic soft materials: magnetically controlled drug delivery, magnetic hyperthermia, MSM‐based scaffolds, and cellular magnetostimulation platforms. (A) A flexible and elastic magnetically responsive nanofiber ceramic scaffold generates a “pump‐like” effect under an alternating magnetic field, squeezing out and releasing encapsulated drugs, Reproduced with permission.[141] Copyright 2022, Bioactive Materials; (B) An implantable biomimetic bone magnet attracts drug‐loaded magnetic nanoparticles circulating in the blood to accumulate in the tumor area, utilizing the magnetothermal effect to achieve drug release, Reproduced with permission.[144] Copyright 2023, Materials & Design; (C) A magnetic scaffold capable of shape recovery triggered by heat or an alternating magnetic field, Reproduced with permission.[147] Copyright 2024, Additive Manufacturing; (D) Wireless magnetostimulation applied by a magnetically responsive hydrogel activates the Ras/MEK/ERK pathway, accelerating healing by promoting cell proliferation and angiogenesis, Adapted with permission [152]. Copyright 2023, Avanced Materials.

Material innovation serves as the core driver of advancements in medical technology. To address the limitations of biological inertness and magnetic particle toxicity in existing materials, Zhang et al. [141]. developed a fully inorganic, flexible magnetic‐responsive nanofiber ceramic scaffold. This material combines bioactivity with a unique “pump” effect: under an alternating magnetic field, it rhythmically compresses to actively expel drugs, achieving on‐demand accelerated release and paving a new path for the design of next‐generation biomaterials, as shown in Figure 4A.

#### 3.2.2 Magnetic Hyperthermia

Embedding magnetic particles into soft matter enables remote thermal ablation of cancer cells by generating heat through AMFexcitation. This therapy has garnered significant attention due to its non‐invasive nature and controllability. In recent years, researchers have focused on theoretical analysis and developing novel magnetothermal composite materials to enhance the efficiency of magnetic hyperthermia.

A key challenge in the clinical translation of magnetic nanoparticles for magnetic hyperthermia is the significant decline in thermal therapy efficiency due to aggregation after cellular uptake. During aggregation, the physical motion of the particles is restricted, while the magnetic dipole interactions generated by close packing also inhibit the flipping of their internal magnetic moments.

Esther et al. [142] innovatively introduced graphene oxide as a dispersion medium in magnetic hydrogels, effectively restoring the suppressed magnetothermal effect. They co‐dispersed PEGylated porous magnetic nanoflowers and Graphene oxide (GO) nanosheets in hydrogel. The study found that GO preferentially interacts with the hydrophobic segments of the hydrogel, thereby physically isolating the magnetic nanoflowers and preventing their aggregation during gelation. This GO‐mediated dispersion significantly weakens the magnetic dipole interactions between the nanoflowers, allowing their Néel relaxation contribution to recover, which markedly enhances the heating efficiency of the composite hydrogel, achieving up to a 23‐fold improvement in equivalent hyperthermia efficiency.

García‐Acevedo et al. [143] developed an in vitro model capable of simulating the intracellular aggregation state of nanoparticles. Their research revealed that in the aggregated state, the linear relationship between coercivity —traditionally considered a key indicator of hyperthermia efficiency— and specific absorption rate (SAR) is disrupted. Instead, a stable and robust linear positive correlation was observed between remanence and SAR. This finding indicates that under high‐density aggregation conditions mimicking the intracellular environment, remanence is a more reliable key parameter than coercivity for predicting and optimizing magnetothermal therapy efficiency, providing new guidance for designing highly efficient magnetic nano‐heaters for clinical applications.

To address the limitations of traditional magnetic targeting therapy—such as reliance on external magnetic fields, high costs, difficulty in precise localization, and potential damage to surrounding tissues—Liang et al. [144] developed an injectable biomimetic bone magnet, please refer to Figure 4B. This system consists of Polymethyl methacrylate (PMMA) bone cement blended with Nd_2_Fe_14_B permanent magnet particles and Fe_3_O_4_ magnetic nanoparticles. Once implanted in the body, the bone magnet generates an internal magnetic field that attracts drug‐loaded magnetic nanoparticles circulating in the bloodstream to accumulate in the tumor area. Subsequently, the magnetothermal effect triggers a liquid‐to‐gas phase transition in the phase‐change material within the drug‐carrying nanoparticles, leading to controlled drug release. Within 100 s, the temperature rises significantly and stabilizes at a high level (close to 60°C) around 150 s.

#### 3.2.3 MSMs‐Based Scaffolds

MSMs has emerged as a powerful class of *active* scaffolds in tissue engineering and regenerative medicine, enabling wireless, spatiotemporally controlled mechanical signaling unattainable with passive biomaterials. Magnetic scaffolds can serve not only as static cell carriers but also dynamically modulate local stiffness, generate cyclic deformation or torque, and induce directed cell migration and differentiation under external magnetic fields, thereby more accurately mimicking physiologically relevant mechanobiological environments.

The seminal work of Garcia‐Gonzalez, Raman, and colleagues established the conceptual framework of magneto‐mechanical scaffolds as platforms for mechanomedicine, where magnetic actuation delivers controllable mechanical stimuli to cells and tissues in a non‐invasive and reversible manner [145]. Their research systematically elucidated how magnetic particles embedded in soft hydrogel or polymer networks transduce magnetic fields into biologically relevant forces, enabling dynamic control over cell fate, migration, and tissue remodeling. Critically, these studies emphasize design principles directly relevant to translational scaffold design, including particle‐matrix coupling, magnetic anisotropy, and force magnitude matching.

Complementing these conceptual advances, Ning et al. integrated high‐resolution 3D bioprinting with magnetic nanomedicine to fabricate a perfusable hydrogel scaffold mimicking the pulmonary vein bifurcation, demonstrating magnetically guided drug delivery under flow within this biomimetic vascular model. This work highlights the feasibility of combining magnetic scaffolds with physiologically realistic geometries for localized, efficient therapeutic delivery [146].

At the device and application level, Hanif and researchers developed a biodegradable magnetic shape‐memory polymer scaffold based on polycaprolactone and Fe_3_O_4_ nanoparticles, which exhibits remote shape recovery under an alternating magnetic field, as shown in Figure 4C. They demonstrated a 3D‐printed tracheal stent prototype, showcasing a complete translational workflow from material formulation and finite element‐guided design to implant‐relevant structures [147].

More recently, magnetically actuated scaffolds have been proposed as soft microrobots for minimally invasive cell delivery systems. For instance, Tian et al. reported alginate‐based magnetic micro‐scaffolds assembled into flexible microrobots capable of magnetic navigation and on‐demand degradation to release mesenchymal stem cells, representing a promising convergence of magnetic scaffolds, soft robotics, and regenerative therapy [148]. Collectively, these studies demonstrate that magnetic soft scaffolds are not merely functional biomaterials but active, remotely controlled therapeutic systems bridging mechanobiology, tissue engineering, and emerging clinical devices.

#### 3.2.4 Cell Mechanostimulation Platforms

Magnetic soft matter provides an ideal wireless medium for delivering physical stimulation to cells, enabling the non‐contact conversion of applied magnetic fields into controllable mechanical signals for precise modulation of cellular behavior. These platforms show unique advantages in cell reprogramming, tissue regeneration, and disease model construction. Their core principle lies in transducing magnetic forces into cellular‐scale stress, strain, or torque signals, which then activate downstream biological processes via mechanotransduction pathways.

Recently, the Garcia‐Gonzalez team has systematically established the theoretical and experimental framework for magneto‐mechanical stimulation platforms, proposing magnetic responsive materials as tools for “mechanomedicine” to deliver programmable mechanical stimuli to cells in vitro and potentially in vivo [149]. Their research shows that magneto‐active polymer substrates or scaffolds can generate dynamic deformation under magnetic fields, which is stably transmitted to cells, triggering cell stiffness remodeling, cytoskeletal rearrangement, and activation of mechanosensitive ion channels.

Advancing direct magneto‐mechanical stimulation, Gómez‐Cruz et al. further developed a cell mechanics platform (the NeoMag system) based on magneto‐active substrates, enabling multi‐modal, reversible cyclic strain application at the single‐cell level. They quantitatively linked the applied stimulation to local changes in cell stiffness and calcium signaling, providing direct evidence for the biophysical mechanisms of magnetic mechanical stimulation [150]. The study found that different loading directions and strain rates lead to distinct cellular mechanical remodeling behaviors, mediated through Piezo1 channel‐regulated intracellular calcium dynamics.

At the molecular‐level of magneto‐mechanical regulation, Del Sol‐Fernández and Garcia‐Gonzalez et al. proposed the MagPiezo magnetogenetics platform. It utilizes high‐anisotropy ferrite nanoparticles (<20 nm) to generate pico‐Newton scale torque under low‐intensity (<40 mT), low‐frequency magnetic fields, enabling wireless activation of endogenous Piezo1 ion channels [151]. This system can induce calcium influx, cytoskeletal rearrangement, and transcriptional activation in endothelial cells without genetic modification, marking a significant leap from “material‐level deformation” to “molecular‐level mechanical regulation.”

In applied research, Shou et al. proposed a “mechano‐activated cell therapy” strategy, as shown in Figure 4D, using cyclic deformation of a magnetic hydrogel to significantly promote cell proliferation and protein secretion for diabetic wound healing [152]. Pan et al. utilized magnetically induced currents to create a wireless electrostimulation scaffold for nerve regeneration [153]. Shen et al. demonstrated that magneto‐piezoelectric nanomotors could generate in‐situ microcurrents in vivo, significantly promoting bone and vascular regeneration [154]. Together, these works show that wireless physical regulation platforms based on magnetic soft matter—whether through direct mechanical deformation or indirect electrical signaling—are forming a new class of cell intervention technologies with high programmability and clinical translation potential.

#### 3.2.5 Biomedical Safety and In Vivo Fate of Magnetic Fillers

Although magnetic soft materials (MSMs) show significant potential in biomedical applications such as drug delivery, magnetic hyperthermia, and tissue engineering, their long‐term in vivo safety and the ultimate biological fate of the magnetic fillers remain critical bottlenecks for clinical translation. Current research primarily focuses on short‐term functional performance, with a notable lack of systematic discussion on the release behavior of magnetic particles following soft matrix degradation and the associated systemic risks.

For the most commonly used magnetic fillers—iron oxide nanoparticles (Fe_3_O_4_, γ‐Fe_2_O_3_)—substantial in vivo studies indicate a degree of biodegradability. These particles can be enzymatically digested within lysosomes via pathways similar to ferritin metabolism, releasing iron ions into the body's normal iron cycle. They are primarily cleared by the mononuclear phagocyte system (MPS), accumulating in organs like the liver and spleen. Extensive animal studies suggest that, within reasonable dosage ranges, iron oxide particles typically induce only transient oxidative stress without causing irreversible organ damage, demonstrating a relatively controllable safety profile [155, 156].

However, for non‐iron‐based magnetic fillers, particularly cobalt‐ and nickel‐based particles (e.g., Co, CoFe_2_O_4_, Ni, NiO), existing research consistently points to significantly higher biotoxicity risks. Cobalt nanoparticles, after phagocytosis and partial dissolution in lysosomes, can induce massive generation of reactive oxygen species (ROS), leading to mitochondrial damage, inflammatory responses, and cell death, potentially even triggering regulated cell death mechanisms like ferroptosis. Systemic exposure studies show that cobalt particles can accumulate in the liver, kidneys, heart, and nervous system, posing risks of multi‐organ toxicity [157, 158]. Similarly, nickel‐based magnetic nanomaterials have been confirmed to exhibit significant cytotoxicity and genotoxicity, primarily through mechanisms including oxidative stress, inflammation, immunotoxicity, and the bioavailability of Ni^2^^+^ ions. Especially under long‐term or high‐dose exposure, nickel‐based particles may cause damage to the respiratory, cardiovascular, and nervous systems, with a safety profile far inferior to that of iron oxide systems [159].

For MSMs, the genuine risk often stems not from the intact composite but from the “release of magnetic fillers after matrix degradation.” Many current strategies encapsulate magnetic particles within hydrogels, elastomers, or biodegradable polymers to achieve good short‐term biocompatibility [160]. However, once the matrix undergoes hydrolysis or enzymatic degradation, the exposed particles may enter the circulatory system and accumulate in MPS organs. While physiological metabolic pathways may partially mitigate this process for iron oxide systems, it could lead to long‐term chronic toxicity risks for cobalt, nickel, or rare‐earth permanent magnet (e.g., NdFeB) systems, for which systematic long‐term in vivo data is currently lacking.

Therefore, from a clinical translation perspective, the biomedical safety of MSMs can be categorized into “partially addressed” and “open” issues. On one hand, the clearance pathways and short‐term safety mechanisms of iron oxide‐based fillers are relatively well‐understood. On the other hand, the long‐term fate, chronic toxicity thresholds, and potential genetic and neurological risks of cobalt‐, nickel‐, and rare‐earth‐based fillers released from degrading soft matrices remain open questions, urgently requiring validation through systematic animal models and long‐term tracking studies. Future design of MSMs must not only focus on magnetic responsiveness but also treat “filler intrinsic toxicity + matrix degradation behavior + in vivo metabolic pathways” as equally critical design constraints.

### 3.3 Flexible Electronics

Flexible electronics technology, recognized globally as one of the top ten cutting‐edge technologies, is revolutionizing traditional rigid circuit architectures with its unique mechanical deformability and providing a robust technical foundation for building a human‐machine‐object integrated intelligent world. This technology enables conformal attachment to complex curved surfaces, demonstrating significant application potential across various high‐tech fields such as intelligent robotics, human‐computer interaction, healthcare, and aerospace.

#### 3.3.1 Flexible Sensors

MSMs provides an ideal platform for fabricating flexible magnetic field sensors and mechanical sensors that can closely conform to complex surfaces. These sensors can be integrated into “electronic skins” for applications in non‐contact human‐machine interaction, motion monitoring, or robotic tactile perception. Current research focuses not only on achieving basic signal detection but also on enhancing the sensors' ability to interpret complex signals, as well as integrating sensing with other functions such as actuation.

**FIGURE 5:**
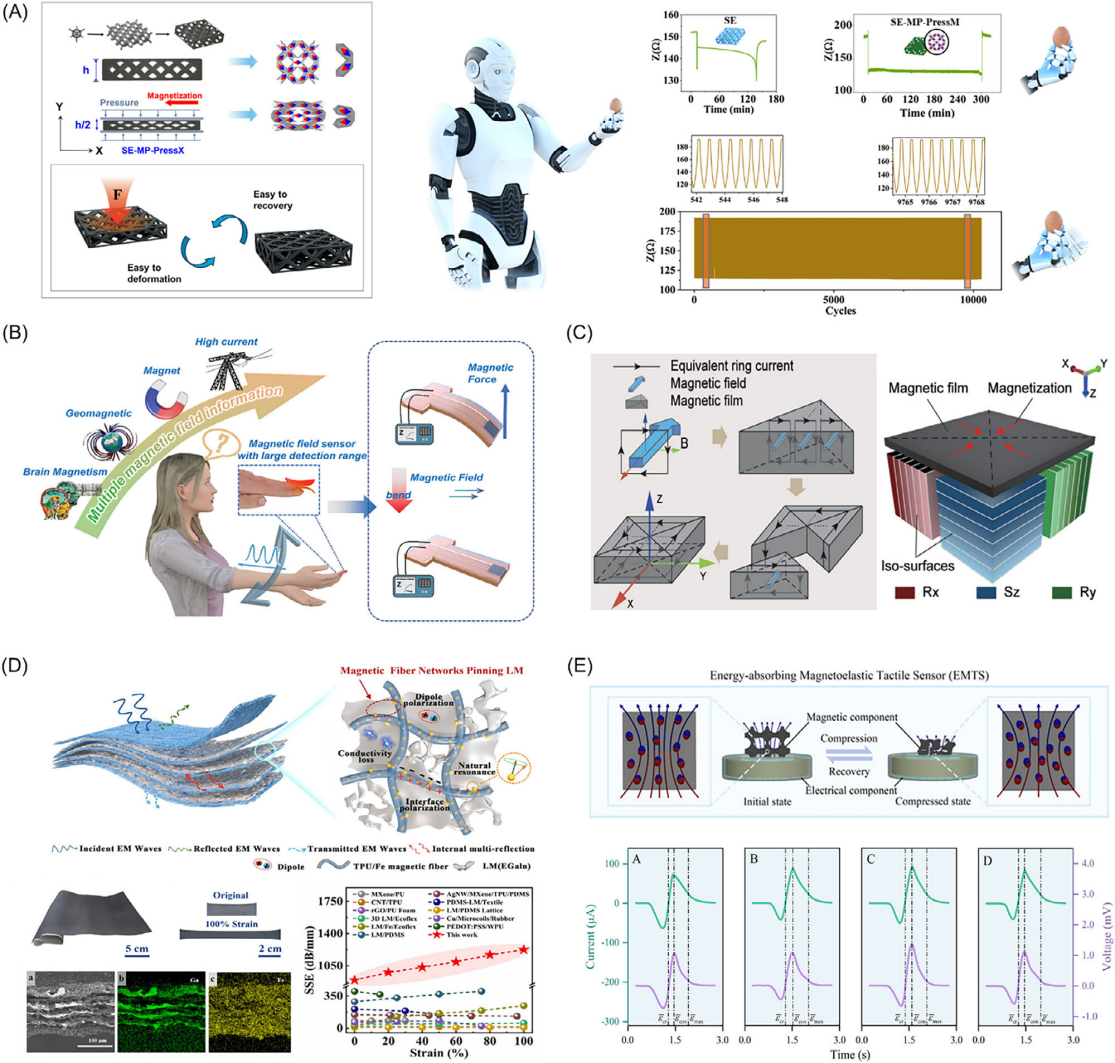
Flexible Electronics Applications Based on Magnetic soft materials: Flexible Sensors, Flexible Electronic Communication, and Flexible Self‐Powered Energy Sources. (A) 3D‐printed topological magnetic network utilizing magnetic pole repulsion to achieve non‐contact repulsive forces, significantly reducing hysteresis and energy loss, providing a novel material solution for high‐precision, long‐lifetime robotic tactile perception, Adapted with permission.[33] Copyright 2025, Iscience; (B) Flexible magnetic sensor enabling wide‐range high‐sensitivity detection by integrating the giant magnetoimpedance effect of amorphous wires with a cantilever structure, Reproduced with permission.[161] Copyrighrt 2023, Advanced Science; (C) Flexible sensor with three‐dimensional force decoupling capability, featuring a tactile layer composed of a centripetally magnetized silicone magnetic film, Adapted with permission.[162] Copyright 2023, Advanced Materials.; (D) Ultra‐thin, stretchable, high‐performance electromagnetic interference shielding film with excellent strain stability, based on magneto‐electric synergistic effects and a pinning‐interlocking mechanism, Adapted with permission.[55] Copyright 2025, Advanced Science.; (E) Magnetoelastic tactile multifunctional sensor, combining self‐powering capability with energy absorption functionality, Adapted with permission.[167] Copyright 2024, ACS Applied Materials & Interfaces.

Traditional elastomers exhibit mechanical hysteresis, energy dissipation, and signal drift, which compromise the long‐term dynamic and static performance of flexible tactile sensors. Existing improvement methods (such as chemical cross‐linking and filler reinforcement) often lead to increased stiffness, embrittlement, or reduced reversibility. Run‐Wei Li's team ingeniously proposed a topological magnetization network structure, which involves magnetizing a 3D‐printed rhombic dodecahedron grid structure under compression [33]. This design causes like magnetic poles to repel each other, generating non‐contact magnetic repulsion forces that significantly reduce viscous dissipation and hysteresis in the elastomer, as shown in Figure 5A. This approach provides a novel material solution for achieving high‐precision and long‐lasting tactile perception in robots. Furthermore, addressing the challenge of simultaneously achieving low detection limits and wide detection ranges in existing flexible magnetoelectronic devices, the team combined the giant magnetoimpedance effect of amorphous wires for low‐field sensing with a cantilever structure integrated with flexible magnetic blocks for high‐field detection. This integrated design enabled a single sensor to achieve high‐sensitivity detection across a broad range from 22 nT to 400 mT, offering a new solution for wearable electronic skins in fields such as navigation, human‐computer interaction, healthcare, and security [161] (Figure 5B).

Enhancing the dimensionality and accuracy of tactile perception relies heavily on sophisticated magnetic design and algorithmic integration. Inspired by human skin and the lateral line system of fish, Peng Zhao's team developed a split‐type magnetically controlled soft tactile sensor [162]. By combining specially designed centripetally magnetized flexible magnetic films with triaxial Hall sensors, they successfully achieved decoupled analysis of three‐dimensional contact forces (Figure 5C). Building on this, the team further created a large‐area wireless flexible magnetic sensor with ultra‐high resolution [53]. This design utilizes multi‐directionally magnetized flexible magnetic films as “skin” – when touched, the underlying sensor array captures unique magnetic field variations. Through deep learning algorithms that analyze these signals, the system not only autonomously distinguishes between pressing and sliding forces but also reconstructs contact point locations with a precision up to 54 times higher than the physical spacing of the sensors. This breakthrough provides a crucial pathway for equipping robots with refined tactile perception capabilities.

In the field of functional integration, researchers are committed to developing multifunctional integrated devices that combine sensing and active regulation capabilities. Sun et al. [163] have created a novel flexible sensor based on conductive magnetorheological fluid(cMRF) that ingeniously utilizes the composite characteristics of cMRF to achieve a unique dual magnetic‐mechanical response: it can not only detect tensile deformation through changes in its own electrical resistance like traditional strain sensors but also actively and reversibly adjust its stiffness in real time with the help of an external magnetic field. This design, which perfectly integrates sensing and actuation functions, opens up new possibilities for developing next‐generation soft robots and wearable devices capable of actively adapting their forms and functions according to environmental changes.

#### 3.3.2 Flexible Electronic Communications

Flexible ferrite sheets and other soft magnetic materials play a crucial role in flexible electronic communication. These materials, characterized by high magnetic permeability and low loss, are used as magnetic shields in antenna devices to prevent internal interference, or for manufacturing stretchable antennas and foldable inductors/transformers.

Developing shielding materials that combine excellent shielding effectiveness with mechanical flexibility is a prerequisite for ensuring signal integrity in flexible devices. In this regard, researchers have made significant progress through composite material design. For example, Zhao et al. [164] constructed a “sandwich” structure of MXene, iron‐nickel alloy, and aramid nanofibers, utilizing a dielectric‐magnetic loss synergy mechanism to achieve an X‐band shielding effectiveness of up to 60.7 dB. In addressing the challenges of dynamic deformation, Zhang et al. [55] developed an ultra‐thin stretchable shielding film with a thickness of only 85 µm. Through a unique magneto‐electric synergistic effect and an internal interlocking mechanism, this film achieved stable shielding effectiveness exceeding 70 dB over an ultra‐wide frequency range from 0.1 MHz to 40 GHz, effectively overcoming the critical challenge of balancing ultra‐thinness, high performance, and dynamic stability in traditional materials, as shown in Figure 5D.

Beyond serving as a “shield” for protection, magnetic or flexible materials play a central role in the design of antennas themselves – the “spear.” One key approach involves leveraging magnetic materials to enhance antenna performance. Carvalho et al. [165] innovatively introduced a Polyvinyl Alcohol(PVA)/Fe_3_O_4_ magnetoactive layer into a paper‐based antenna via screen printing. This magnetic layer effectively concentrates electromagnetic flux, significantly optimizing the antenna's reflection coefficient from −8.9 dB to −55.8 dB and markedly increasing the communication distance, thereby paving a new path for developing low‐cost, high‐performance flexible antennas.

Another core challenge is ensuring the functional stability of antennas under extreme deformation. To address this, He et al. [54] developed a highly stretchable near‐field communication antenna based on a bio‐inspired spiderweb structure and liquid metal. Its sophisticated structural design overcomes the critical weakness of traditional flexible antennas, whose resonant frequency tends to shift under mechanical deformation, enabling stable communication performance even under 300% tensile strain and severe twisting. The antenna maintains functional stability under folding (0–170°), bending (radius 2.5–20 mm), twisting (0–270°), rolling (0–360°), and overall pressure (1000 kPa), laying a solid foundation for wireless interaction in wearable devices and soft robots.

#### 3.3.3 Flexible Self‐Powered Devices

Harnessing the physical effects of soft matter for energy harvesting represents a significant transformation of flexible electronics from relying on external power sources to achieving energy self‐sufficiency. In this field, MSMs plays a key role due to its unique magneto‐mechano‐electric coupling characteristics. Currently, there are two main technical pathways: one utilizes the magnetoelastic effect to directly convert mechanical energy into electrical energy, and the other integrates magnetic materials with other power generation mechanisms to enable richer functional integration.

Based on the magnetoelastic effect, energy harvesters represent a mainstream direction in this field, with their core principle focusing on efficiently converting biomechanical motion into electrical energy. The research team led by Professor Jun Chen at the University of California, Los Angeles, has developed a mass‐producible textile‐based magnetoelastic patch [166]. This device utilizes deformation caused by muscle movement to alter its internal magnetic field, which then induces an electrical signal through coil induction, achieving self‐powered sensing. With high stretchability, high sensitivity, and excellent durability, it can accurately monitor data such as arm bending and grip strength, perfectly demonstrating the significant potential of magnetoelastic energy harvesters as self‐powered sensors for wearable devices.

Building upon the core mechanism, structural innovation can impart additional functionalities to devices. A prime example is the soft magnetoelastic tactile multi‐functional sensor developed by Lin et al. [167] Instead of adopting a conventional solid structure, they designed a periodic porous metamaterial based on Fourier series. This sophisticated structure not only enhances energy conversion efficiency through the buckling effect but also achieves an energy loss coefficient as high as 49.7%, enabling the device to serve dual functions of self‐powered sensing and efficient impact absorption, as shown in Figure 5E.

Integrating magnetic materials with different power generation mechanisms enables higher‐dimensional functional integration, representing one of the most forward‐looking development directions in this field. The magnetic sponge triboelectric nanogenerator developed by Li et al. [168] achieves a trinity of energy harvesting, sensing, and drug delivery. While utilizing triboelectricity as its primary energy source, the clever incorporation of magnetic particles and liquid metal allows the device to not only function as a self‐powered force/magnetic field sensor but also, under external magnetic control, act like a miniature soft robot to achieve controlled drug release in a simulated gastric environment. This research provides an exceptionally important conceptual framework for developing next‐generation multifunctional flexible devices capable of “energy self‐sufficiency, intelligent perception, and active execution.”

### 3.4 Energy and Environment

#### 3.4.1 Energy Harvesting and Storage

Widely available low‐frequency mechanical energy, acoustic energy, and wind energy in the environment, though limited in energy density and difficult to utilize directly, can be efficiently converted into electricity through novel materials and structural designs. This not only provides sustainable power for small electronic devices but also opens new pathways for green energy development and the deployment of intelligent systems. In recent years, researchers have proposed a series of innovative energy harvesting strategies based on MSMs and flexible composite materials, demonstrating remarkable energy conversion efficiency and application potential.

**FIGURE 6:**
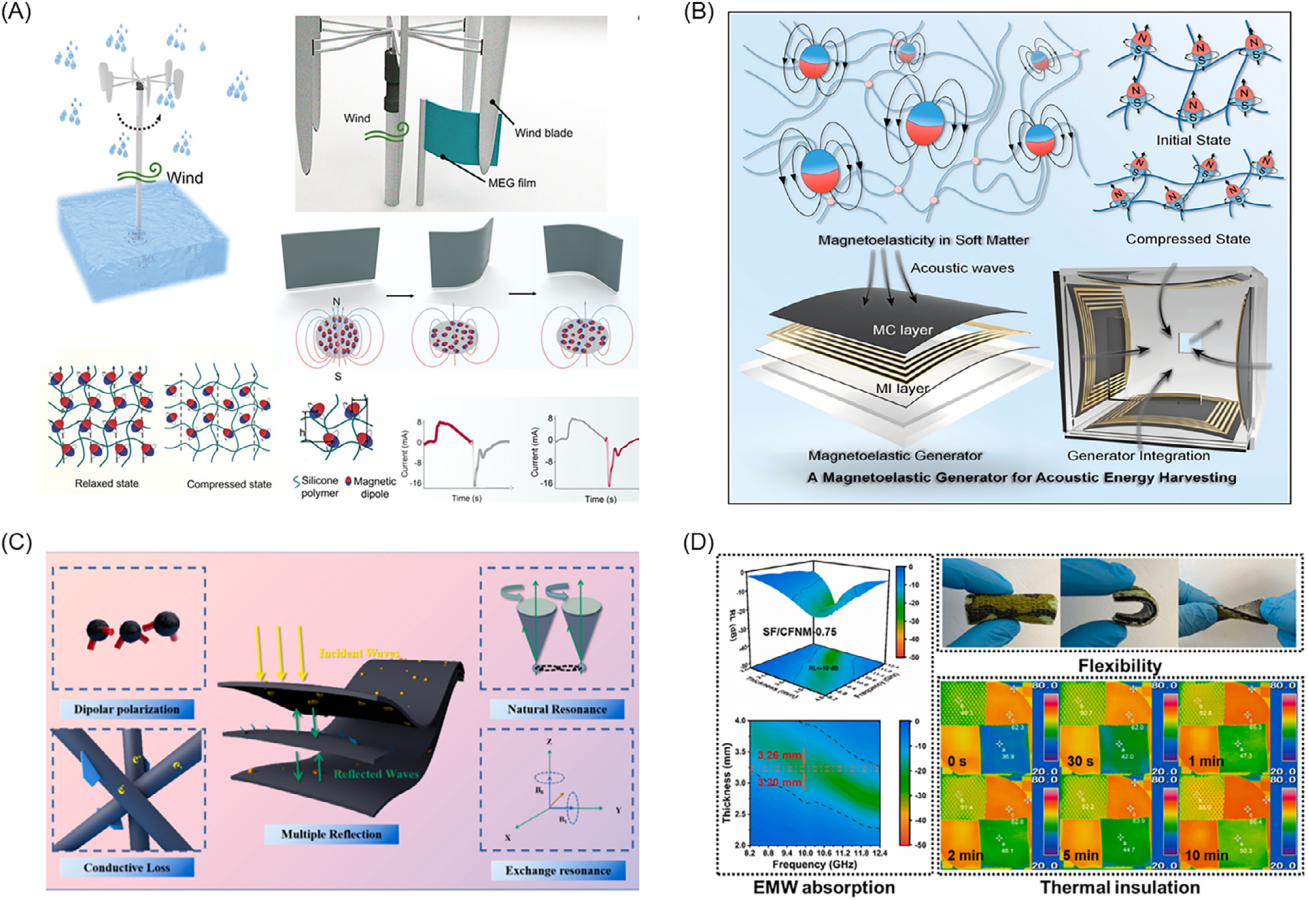
Environmental energy harvesting & storage, absorption & dissipation based on Magnetic soft materials. (A) Schematic of a flexible magnetoelastic energy harvester: wind‐induced deformation alters magnetic flux, inducing current in the coil and enabling power output at the mW/cm^2^ level, Reproduced with permission.[169] Copyright 2022, Advanced Materials. (B) An acoustic energy harvester utilizing the giant magnetoelastic effect directly converts sound vibrations into electricity, achieving a hundredfold performance enhancement over existing technologies, Reproduced with permission.[56] Copyrght 2025, Matter. (C) A sandwich‐structured film composed of a carbon nanofiber framework, MoS_2_ dielectric layer, and CoFe_2_O_4_ magnetic layer achieves perfect integration of microwave absorption and thermal conduction, Reproduced with permission.[172] Copyright 2025, Carbon. (D) A flexible magnetic wave‐absorbing material, designed via multi‐scale synergy, achieves full X‐band absorption and thermal stealth within a broad thickness range of 3.20–3.26 mm, Reproduced with permission.[173] Copyright 2023, Carbon.

The Jun Chen team developed a groundbreaking flexible magnetoelastic energy harvester [169], as shown in Figure 6A. The team designed a magnetoelastic film composed of permanent magnet micro‐particles uniformly dispersed in a flexible elastomer. The core innovation of this work lies in the clever utilization of the giant magnetoelastic effect in soft materials: when a gentle breeze passes, the highly deformable flexible film undergoes rapid fluttering or vibration, and this mechanical deformation directly alters the internal magnetic field distribution of the material. According to Faraday's law of electromagnetic induction, this rapid change in magnetic flux induces a sustained current in an externally wound coil, thereby efficiently converting wind energy into electrical energy. The device can generate a power density as high as 0.82 mW/cm^2^ under ambient wind conditions, capable of not only powering small electronic devices but also driving water electrolysis to produce hydrogen. This research provides a novel, efficient, and scalable technical solution for harvesting low‐frequency mechanical energy using MSMs.

Subsequently, the Jun Chen team applied the giant magnetoelastic effect in soft matter to acoustic energy harvesting [56], as shown in Figure 6B proposing an Acoustic Energy Harvester based on this effect, designed to efficiently capture energy from ambient sounds. Its operating principle relies on the giant magnetoelastic effect: sound waves cause film vibrations, leading to the rearrangement of magnetic particles within the elastic matrix, which alters the magnetic field and generates an induced current in the coil. This AEH achieves a short‐circuit current density of 98 µA/cm^2^ with an internal resistance of only 300 Ω, outperforming existing acoustic energy harvesters by 100 times. It operates effectively across a frequency range of 0–900 Hz, covering various sound sources. The device can withstand 142% tensile strain and maintains performance after over 90 000 cycles without degradation.

Compared to energy harvesting, the application of MSMs in the energy storage field is still in its early stages, but its reversible deformation and magnetic responsiveness enable novel energy storage mechanisms. Inspired by octopus suckers, Wang's team developed a magnetoelastic soft actuator [170]. Its core mechanism involves using magnetic fields to gradually deform an elastic membrane for potential energy storage, followed by instantaneous energy release upon magnetic field removal to drive cavity‐generated negative pressure for rapid and strong adhesion. This strategy of converting magnetic energy into controllable elastic potential energy provides new insights for developing efficient soft gripping and reversible adhesion systems.

#### 3.4.2 Energy Absorption and Dissipation

Addressing the core challenge of “low filler content vs. high wave absorption efficiency” in electromagnetic energy management, researchers have proposed a series of solutions through structural innovation:

Yu's team utilized magnetic field‐induced alignment of carbonyl iron powder in elastomers to form ordered chain‐like structures, constructing efficient magnetic/electrical loss networks at a low filler content of 40 wt.%, significantly enhancing impedance matching and electromagnetic dissipation efficiency [57]. Zhou et al. adopted a synergistic strategy combining macroscopically 45° stacked carbon cloth with vertically grown NiCo_2_O_4_ nanoneedle arrays, expanding the absorption bandwidth from 2.24 to 7.44 GHz [171]. Huang's group developed a sandwich‐structured composite film(Figure 6C) that simultaneously attained 7.8 GHz absorption bandwidth and 0.163 W/(m·K) thermal conductivity [172]. Chen's team achieved full X‐band absorption within a 3.2 mm thickness through a porous aerogel structure(Figure 6D), additionally incorporating thermal stealth properties [173].

These studies break through the limitations of traditional materials via multi‐scale structural design, providing new paradigms for electromagnetic protection in flexible electronic devices.

#### 3.4.3 Environmental Sensing and Monitoring

MSMs, with its unique magnetic properties, is pioneering new pathways in the field of passive environmental monitoring. From employing precisely designed micro‐nanorobots to actively degrade persistent pollutants in water, to ingeniously utilizing naturally available biological carriers for the passive capture and monitoring of magnetic particulate contaminants in the air, researchers are continuously expanding the application boundaries of magnetic materials in environmental science.

To address the challenge of treating refractory organic pollutants in water bodies, Pumera's team developed an intelligent microrobot that integrates magnetic propulsion [174], pollutant adsorption, and photocatalytic degradation. Using chitosan hydrogel as a substrate, the robot simultaneously carries iron oxide and zinc oxide nanoparticles. After precise magnetic navigation to contaminated areas, it first enriches pollutants through the hydrogel, then achieves in‐situ degradation via photocatalytic reactions. This recyclable, active treatment solution offers new insights for water treatment technologies.

To achieve large‐scale, low‐cost monitoring of fine particulate matter pollution in urban air, Chaparro et al. [175] pioneered an innovative method using trees for in‐situ magnetic biomonitoring. The core concept of this study leverages the fact that many PM particles from traffic and industrial emissions in cities contain strongly magnetic iron oxides. These magnetic particles naturally adhere to and accumulate on the rough bark of street trees. The team innovatively demonstrated that by using a portable magnetic susceptibility meter to conduct non‐destructive measurements of tree bark magnetism on‐site, the level of adsorbed PM pollution can be quickly and accurately quantified. This study ingeniously transforms trees into natural, long‐term atmospheric samplers, providing a completely new monitoring approach for mapping urban air pollution distribution and environmental quality assessment that requires no sampling, is non‐destructive, and cost‐effective.

## 4 Challenges and Future Perspectives

### 4.1 Fundamental Theoretical Models: From Phenomenology to Predictive Design

The core bottleneck for MSMs lies not in a “lack of models,” but in the systematic failure of classical assumptions under conditions of ultra‐softness, large deformation, and dynamic magnetic actuation. Existing continuum models still struggle to reliably predict performance under dynamic fields, finite strains, and the coupling of particle interactions with matrix viscoelasticity [84, 176, 177, 178]. Moreover, many critical parameters are difficult to isolate experimentally, thereby hindering the leap from phenomenological description to predictive design.

Key failures are concentrated in three categories:

1. Failure of the Small‐Strain Assumption: Ultra‐soft systems and magnetically driven structures often experience strains ranging from tens to hundreds of percent, rendering linear magneto‐elastic constitutive laws insufficient for describing the true response. To address this, finite‐deformation magneto‐visco‐hyperelastic continuum models have been established with thermodynamic consistency and numerical implementation, providing a usable framework for large deformations and coupled dissipation [154].
2. Failure of the Quasi‐Static Magnetics Assumption: Under AMFactuation, strong coupling between magnetic fields and structural dynamics exists, where the type of magnetic source and boundary conditions determine the local magnetic stress distribution and material response. Incorporating realistic magnetic boundaries and fully coupled solvers can significantly enhance predictive capability [179].
3. Failure of the Affine Particle Rotation Assumption: Non‐affine rearrangements are prevalent in high‐filler‐content/strongly coupled systems, potentially inducing macroscopic anisotropy and mode transitions. Coupled pathways of explicit microstructure modeling and homogenization can quantitatively reveal the competition between microstructural rearrangement and macroscopic deformation [180].

The boundary between “solved” and “unsolved” problems at this stage is also clearer: a usable toolchain comprising finite‐deformation magneto‐viscoelastic continuum theory, multi‐scale homogenization, and full‐field finite element methods with realistic magnetic boundaries has been established. However, three categories of open problems remain:

1. Residual magnetization‐induced implicit anisotropy may lead to qualitative errors in topology/inverse design [89].
2. Whether micropolar/Cosserat theory must be introduced to characterize couple stresses and additional rotational degrees of freedom under strong magnetic torques and local bending [181].
3. The most critical contradiction between model fidelity and computational tractability (difficult parameter identification and high computational cost) still hinders the practical implementation of topology optimization, real‐time control, and inverse design.

Therefore, the future direction should shift from “adding degrees of freedom” to achieving “controllable fidelity.” Physics‐Informed Neural Networks (PINNs) and multi‐scale frameworks can integrate sparse experimental data under the constraints of conservation laws to inversely identify constitutive parameters. Surrogate models trained on simulation data can replace expensive FEM in design iterations, enabling near‐real‐time optimization and closed‐loop design.

### 4.2 Advanced Manufacturing: Bridging the Gap From Lab‐Scale to Functional Systems

The core contradiction in MSM manufacturing is the inherent difficulty in simultaneously achieving magnetic microstructure controllability, high‐precision magnetization distribution, and scalable production capabilities [182, 183, 184, 185]. Current major obstacles remain concentrated in 3D orientation control, the manufacturability of continuous magnetization, and process conflicts in multi‐material/complex structures.

First, high‐resolution orientation control in three‐dimensional space is constrained. Magnetic field‐assisted printing/curing can induce alignment, but high filler loading leads to increased viscosity and enhanced inter‐particle magnetic coupling, causing alignment response hysteresis and reduced stability. Even in systems with high filler content (∼50 vol%), it is challenging to balance fine orientation control with the design freedom for complex 3D structures simultaneously [186].

Second, the theoretically designable continuous magnetization field is difficult to be “faithfully realized” in manufacturing. While trajectory encoding in Direct Ink Writing (DIW) can achieve a degree of continuity, it heavily relies on path‐generation algorithms and the precision of local field control. Existing slicing/path planning tools struggle to automatically map the continuous magnetization distributions obtained from topology optimization into printable paths. This results in most physical prototypes remaining at the level of discrete magnetization units [187].

Third, significant process conflicts persist for multi‐material and complex overhanging structures. The strong light absorption by magnetic particles reduces photopolymerization efficiency, worsens interfacial bonding, and compromises structural accuracy. Under high filler loading conditions, incomplete cross‐linking and interfacial defects increase markedly, leading to a significant rise in process complexity [188].

Despite this, technologies such as pixel‐level in‐situ magnetic programming and multi‐axis electromagnetic arrays have pushed the boundaries of magnetization freedom and structural complexity to a higher level. However, they generally rely on customized hardware and control algorithms, resulting in high costs and difficulties in scaling up [189].

In summary, the key to engineering translation is no longer incremental improvements in individual processes, but the development of integrated, closed‐loop manufacturing platforms. Only by coupling magnetic field control, structural printing, and online feedback calibration into a unified system can we bridge the gap from laboratory prototypes to replicable, functional devices.

### 4.3 Intelligent Material Design: Navigating the Vast Parameter Space

The design space of MSMs is a typical “high‐dimensional, strongly coupled system”: the output behavior is jointly determined by matrix stiffness, filler type/size/content, spatial distribution and magnetization pattern, geometric topology, and external field loading. Traditional trial‐and‐error methods are inefficient under multi‐objective constraints (e.g., large deformation + self‐sensing), making AI‐driven inverse design the future mainstream approach [190, 191, 192].

Current optimization methodologies exhibit a three‐tiered structure:

1. Physics‐Driven Continuous Optimization: This approach centers on finite deformation magneto‐mechanical models, utilizing gradients and sensitivity analysis for the co‐optimization of topology and magnetization. It offers strong physical interpretability but incurs extremely high computational costs [193]. Extending this by integrating the Material Point Method (MPM) enables co‐design under conditions of large deformation, dynamic contact, and time‐varying external fields. However, this further increases the computational burden, making it difficult to scale to large‐scale parameter searches or real‐time design [194].
2. Physics‐Machine Learning Hybrid (Surrogate / Physics‐Informed):Machine Learning (ML) models are used as surrogates for high‐fidelity simulations, significantly reducing computational costs. These models can perform both rapid forward prediction and inverse geometry generation (achieving high R^2^ accuracy) [195]. Concurrently, model‐driven ML can identify optimal constitutive forms and parameters while preserving physical consistency, thereby enhancing modeling and optimization efficiency [196].
3. Purely Data‐Driven Inverse Design: This method directly learns the mapping between “target function” and “structure/magnetization,” offering a clear advantage in exploring non‐intuitive solutions. However, its generalizability and interpretability are highly dependent on the data distribution, limiting its application in safety‐critical scenarios [197].

Therefore, the future paradigm is not about “replacing physics with ML,” but about constructing closed‐loop, self‐optimizing systems. This involves unifying physical constraints, simulation‐generated data, high‐throughput experimental/robotic validation, and manufacturing feedback into a single design cycle. This shifts the core question from “What deformation will this magnetization pattern produce?” to “How should magnetization and material be distributed to achieve this specific task?”

### 4.4 Towards Integrated Actuation‐Sensing and Closed‐Loop Autonomy

A unique, underexploited advantage of MSMs is their intrinsic capability for both actuation and sensing within a single, monolithic material. This enables the development of embodied intelligence and closed‐loop control without relying on bulky, external sensor arrays.

Future Perspective: The pathway to autonomous MSM devices lies in fully exploiting intrinsic sensing modalities. Three key approaches are:

1. Self‐Sensing Actuators via the Magnetoelastic Effect: The same hard‐magnetic domains that drive actuation also generate a measurable magnetic flux change when deformed. By monitoring this flux (e.g., with embedded micro‐coils), the actuator can serve as its own strain/force sensor, enabling real‐time shape estimation and collision detection.
2. Embedded Sensing via Magnetoresistive Effects: Integrating thin films or patterns of GMR/AMR/TMR sensors directly onto or within the MSM structure can provide high‐sensitivity, localized measurement of magnetic field changes—either from external sources or from the material's own deformation—facilitating precise navigation and interaction sensing.
3. Self‐Powered Closed‐Loop Systems via the Magnetoelectric Effect: Harvesting energy from actuation‐induced deformation or ambient vibrations using magnetoelectric composites can power on‐board microcontrollers and sensors. This creates fully autonomous, untethered systems capable of sensing, processing, and responding to their environment without external power or control wires.

## 5 Conclusion

The journey of MSMs from fascinating laboratory prototypes to robust, functional technologies hinges on overcoming the intertwined challenges of predictive modeling, scalable manufacturing, and intelligent design. The future of this field is not merely in improving individual materials, but in engineering tightly integrated “material systems” that seamlessly combine actuation, sensing, computation, and power. This will require deeper convergence with robotics, computer science, and electrical engineering. By embracing AI for design, innovating hybrid fabrication, and leveraging intrinsic multifunctionality, the next generation of MSMs will transition from being remotely operated novelties to becoming adaptive, intelligent, and autonomous entities capable of sophisticated tasks in healthcare, environmental monitoring, and human‐machine symbiosis.

## Author Contributions

Conceptualization, Z. X., B.D., and R‐W.L. investigation, J.S. and R‐W.L. writing – original draft, Z. X., X.X., and Y.Y. writing – review and editing, Z. X., X.X., B.D., and R‐W.L. funding acquisition, R‐W.L. resources, Z. X., X.X., and Y.Y. supervision, R‐W.L. Investigation, B.D.

## Conflicts of Interest

The authors declare no conflicts of interest.

## Data Availability

The authors have nothing to report.
